# Influence of Environmental Covariates on Pollinator Community Occupancy, Detection, and Richness Across Urban Gardens in Richmond, Virginia, USA

**DOI:** 10.1002/ece3.72502

**Published:** 2025-11-17

**Authors:** Nicholas J. Ruppel, Robert B. Nipko, Mackenzie Dingus, Allison C. Ortiz, Teresa Weir, Marcella J. Kelly, Stephanie S. Coster

**Affiliations:** ^1^ Department of Biology Randolph‐Macon College Ashland Virginia USA; ^2^ Department of Fish and Wildlife Conservation Virginia Tech Blacksburg Virginia USA

**Keywords:** *Liatris spicata*, multispecies occupancy models, pollination, *Pycnanthemum muticum*, urban ecology

## Abstract

Pollination is a vital ecosystem service essential for the reproduction of most flowering plants. However, pollinators, especially insects, are in dramatic worldwide decline, threatening food security and global biodiversity. Urban areas, when managed well, can act as critical corridors and reserves for pollinators, benefiting ecosystems beyond the urban environment. This study assessed plant–pollinator interactions in urban gardens across the Mid‐Atlantic (USA) region, focusing on two native plants: dense blazing star ( *Liatris spicata* ) and clustered mountain mint ( *Pycnanthemum muticum* ). Over 350 visual surveys in 52 gardens recorded 361 pollinator detection events involving 14 taxa, with five taxa dominating the detections. Using multispecies occupancy models (MSOMs) in a Bayesian framework, we evaluated how site and survey factors influenced species occupancy, detection, and richness. Species occupancy ranged from 0.04 ( *Halyomorpha halys* ) to 0.86 (Halictidae), with little influence from external variables. Detection was influenced by plant species, survey start time, Julian date, and urban distance. For instance, honey bees ( *Apis mellifera* ) and wasps (Vespoidea) were more likely to visit *P. muticum* , while *Atalopedes campestris* favored *L. spicata* . Detections of Halictidae increased in urban areas, while *Bombus* spp. were more common in rural settings. Our study demonstrates that MSOMs can be an effective tool for monitoring and investigating the urban pollinator community. We were able to estimate occupancy for the 14 observed insect taxa, nine of which were detected fewer than eight times. We also estimated effects of detection covariates that impacted multiple taxa and provided insight into ways to improve future pollinator monitoring efforts. These findings further our understanding of how plant species and the urban setting may variably influence pollinator activity and highlight the importance of urban gardens in supporting diverse insect communities.

## Graphical abstract

Urban gardens can play a vital role in supporting pollinator diversity and aiding ecosystem health amidst global declines in pollinators. This study analyzed plant–pollinator interactions in Mid‐Atlantic (USA) urban gardens using multispecies occupancy models, focusing on two native plants: *Liatris spicata* and *Pycnanthemum muticum* . Findings revealed species‐specific occupancy patterns, detection influenced by plant type and urban distance, and the importance of urban gardens in fostering diverse pollinator communities.

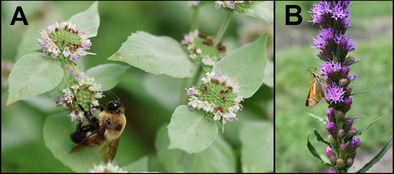

## 1 Introduction

Pollination, facilitated by plant–pollinator interactions, is a vital ecosystem service that supports the reproduction and propagation of most flowering plants, while simultaneously providing sustenance to pollinators. Given the dramatic decline in pollinators worldwide (Goulson et al. 2015), and urban development increasing at unprecedented rates (United Nations Department of Economic and Social Affairs 2023), supporting pollinators with optimal habitat is of utmost importance to the long‐term sustainability of our food supply and seminatural ecosystems. Recent global pollinator declines have spurred investigation into the potential for urban “oases” such as community green spaces, median plantings, and residential gardens to provide resources and habitat for both native and non‐native pollinators (Silva et al. 2023). Ongoing research suggests that urban green spaces can not only provide a stable habitat for native pollinators (Hennig and Ghazoul 2012; Hall et al. 2017), but that urban areas worldwide may support highly diverse populations of both generalist and specialist insect pollinators (Hennig and Ghazoul 2012; Baldock et al. 2015).

One key factor driving the establishment of diverse, urban pollinator populations is the availability of floral resources (Hennig and Ghazoul 2012; Cariveau and Winfree 2015). In particular, native plants play a vital role in supporting native pollinators due to coevolved relationships that create a mutual dependency between species (Kearns et al. 1998; Bartomeus et al. 2013; Emer et al. 2016). Some native pollinators exhibit dietary specialization, relying on particular plant species or genera, while others are more generalist in their foraging (Brosi 2016; Armbruster 2017). Thus, promoting native plants in suburban and urban green spaces helps provide essential foraging resources and habitat, supporting the persistence and resilience of native pollinator communities.

Generally, a wide variety of pollinators occupy the available green spaces in urban or suburban settings. Bees and other insects in the order Hymenoptera often comprise the bulk of pollinators, although members from Coleoptera, Diptera, and Lepidoptera are also commonly present and active (Zaninotto et al. 2021). Urban bee communities, in particular, can be quite robust and diverse (Normandin et al. 2017), with some reports indicating a higher level of support in urban relative to more rural environments (Deguines et al. 2012; Kaluza et al. 2016; Theodorou et al. 2017). These and additional studies have demonstrated that urban ecosystems can be used for pollinator conservation efforts if the right network (e.g., high floral diversity, garden size) of local and regional landscape features is present (Ayers and Rehan 2021).

Despite a growing body of work, there is currently limited information regarding environmental and spatial factors broadly driving occupancy and detection of urban and suburban pollinator populations. This is due in part to the wide‐ranging requirements of individual pollinator species, as different pollinators are drawn to a variety of plants and habitats found in many different regions, and are tolerant to a range of different conditions (Rader et al. 2012; Emer et al. 2016). To develop a more robust dataset of influential factors affecting pollinator distribution, richness, and detectability, we established a study to monitor species richness in public and private green spaces in the urban and suburban environs of a growing city, Richmond, Virginia, USA. From 2010 to 2022, the human population increased by 11% in the city of Richmond and neighboring counties (US Census Bureau 2022). Although development linked to population growth can result in habitat fragmentation and noted declines in pollinator abundance and diversity (Ahrné et al. 2009; Bates et al. 2011), research suggests some urban areas support diverse pollinator assemblages (Fetridge et al. 2008; Matteson et al. 2008; Osborne et al. 2008). In this study, we focused on key site‐ and survey‐level covariates known to affect pollinator richness and abundance. Specifically, we examined the influence of distance to urban center and total garden area, hypothesizing that pollinator responses would vary among taxa along the urban gradient (Ahrné et al. 2009; Williams and Winfree 2013; Udy et al. 2020), and that larger gardens would support greater richness (Baldock et al. 2019). At the survey level, we included garden bloom richness, Julian date, temperature, light level, and time of day, based on evidence that these variables impact pollinator behavior and detectability. For instance, higher bloom richness (Schmack and Egerer 2023), warmer temperatures (Papanikolaou et al. 2017), and greater light availability (McKinney and Goodell 2010) are generally associated with increased pollinator activity, while detection may also vary by time of day (Ruppel et al. 2019) and season (Rafferty et al. 2015; Gallagher and Campbell 2020), depending on the taxa. These covariates allowed us to model occupancy and detection probabilities with greater ecological relevance across urban garden sites.

Understanding plant–pollinator interactions, especially in an urbanizing landscape, is complex and requires the ability to distinguish local and landscape factors. In essence, a predictive methodology for establishing the requirements for cultivating plant communities capable of supporting diverse and stable pollinator populations is key to successfully securing functional refugia for insect pollinators—especially those coadapted to take advantage of native resources (Frankie et al. 2002; Hanley et al. 2014). To do this, we used a Multispecies Occupancy Modeling (MSOM) approach. MSOMs are a class of hierarchical models for examining the structure and distributions of an ecological community (Dorazio and Royle 2005; Kéry and Royle 2016b; MacKenzie et al. 2018). Like other occupancy models, MSOMs account for imperfect detection by incorporating replicate surveys (temporal or spatial) at study sites, and by explicitly defining distinct (but conditionally linked) models for the observation process and the ecological state process (MacKenzie et al. 2002, 2018). By treating species‐specific occupancy and detection probabilities (and possibly covariates) as random effects from community‐level distributions, MSOMs provide an efficient framework for simultaneously estimating parameters for multiple species. Because species‐specific parameters are considered realizations of community‐level random variables, information is shared across species, potentially allowing for stronger inferences even for species with small sample sizes. MSOMs are typically implemented in a Bayesian modeling framework and, with data augmentation, may also incorporate an additional hierarchical level, the supercommunity process, which allows estimation of the total number of species that may have been present in the region (i.e., gamma diversity; Whittaker 1960), even when some species went completely undetected (Dorazio and Royle 2005; Dorazio et al. 2006; Kéry and Royle 2016b).

Our primary objectives were to identify predictors of occupancy and detection probabilities for multiple pollinator species (i.e., predictors that impacted the larger pollinator community, not just individual species), and to estimate the magnitudes of those effects using a model‐based approach. Secondarily, we sought to use the most parsimonious model incorporating those covariates to make inferences about pollinator diversity at our sample sites, and species‐specific occupancy across sites, after accounting for imperfect detection. Extending predictions or inferences to locations we did not sample was beyond the scope of the current analysis. Insights into community‐level drivers of pollinator occupancy have broader implications for land‐use policy and can inform recommendations for managing native pollinator populations, while insights into drivers of detection probabilities may inform study designs to improve future efforts to monitor pollinators.

## 2 Materials and Methods

### 2.1 Monitoring Techniques

**FIGURE 1:**
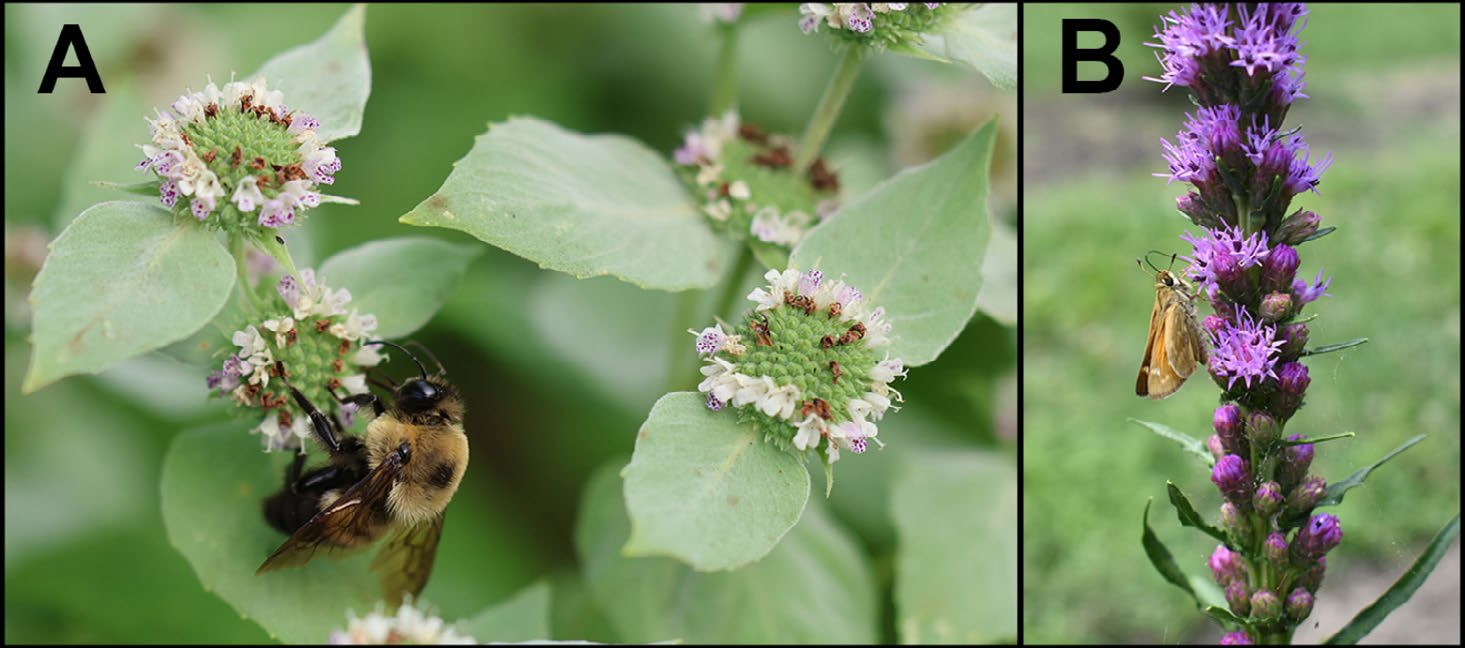
Photographs of floral visitors interacting with the two focal plants, *Pycnanthemum muticum* (A) and *Liatris spicata* (B).

*Liatris spicata* and *Pycnanthemum muticum* were chosen as the focal species because they are regionally native, commonly cultivated in urban green spaces, and considered pollinator friendly (i.e., resource rich) (Fuccillo Battle et al. 2021). *P. muticum* is a perennial plant with flowers growing in dense clusters at the top of stems or just below them, often nestled in the upper leaf joints. Surrounding the flowers are pale, leaf‐like bracts, all of which have a soft, white appearance. The small sepals (2.6–4.2 mm) are nearly symmetrical, with small narrowly triangular lobes at the tips that come to a point. The petals are also small, ranging from 3 to 6 mm (Weakley et al. 2012; Figure 1A). The flower spikes of the perennial *L. spicata* can range from 6 to 70 cm tall, with flower heads that are loosely to densely packed along the stem. Each rounded flower head is about 1 cm wide. Each flower head contains 4–12 small purple florets, the floral tubes of which are smooth on the inside, with the lobes at the tips 2–4 mm long (Weakley et al. 2012; Figure 1B).

**FIGURE 2:**
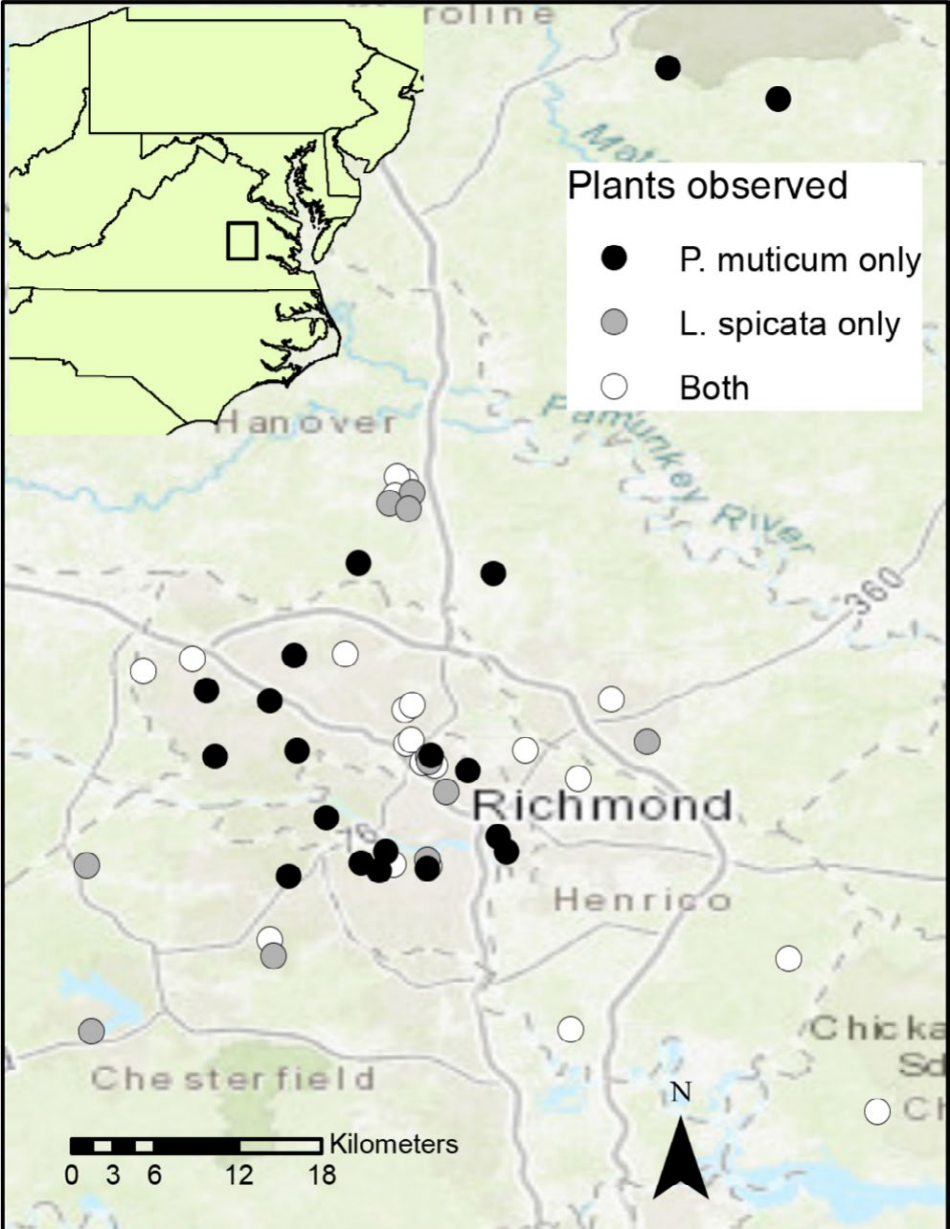
Pollinator monitoring field site locations throughout the greater Richmond, Virginia, USA region where visual surveys were conducted in 2021. Survey locations included those where only *Pycnanthemum muticum* was surveyed (black circles), only *Liatris spicata* was surveyed (gray circles), or both were surveyed on separate occasions (white circles).

The study sites included private and community spaces throughout the greater Richmond, Virginia, USA area (Figure 2, Table A1.1 in Supporting Information), and were opportunistically chosen when landowners that cultivated *L. spicata* and/or *P. muticum* volunteered garden access. The average distance between sites was approximately 20.1 km, with the minimum distance between two sites being 0.16 km. While some individual pollinators might visit multiple sites, leading to some lack of independence for sites in close proximity, the potential for bias is low given that we surveyed such sites within the same day or two (is this correct?), thus it is unlikely that the same individuals would occur in the same sites at the same time/day we visited sites. We assessed *L. spicata* and *P. muticum* plant–pollinator interactions from June 28 to July 27, 2021, resulting in a total of 350 monitoring surveys across 52 sites (two of these sites were subsequently excluded due to missing covariate data). *L. spicata* , which blooms earlier in most locations, was surveyed between June 28 and July 11, 2021; *P. muticum* was surveyed from July 9 to 27, 2021. Although our sampling window did not consist of the entire flying season, this period was chosen because it corresponds with peak bloom for our focal plant species in the Mid‐Atlantic region. By focusing on peak bloom, we captured a representative period of plant–pollinator interactions essential for addressing our study's objectives.

Of the 50 survey sites, 20 included surveys on both plant species, 20 were surveyed only on *P. muticum* , and 10 were surveyed only on *L. spicata* , for a total of 350 surveys (counting spatial replicates) (Table A1.1 in Supporting Information). Each flower species was monitored at separate times for sites that contained both species.

For visual monitoring at each site, five separate inflorescences on different plants (spatial, rather than temporal, replicates) were monitored for insect interactions according to previous protocols (Frankie et al. 2002; Ruppel et al. 2019). Each site was visited once for monitoring, except in cases where the site had both plant species present, in which case each focal species was monitored on separate dates. As each garden space had a different layout, the five plant replicates within each garden were chosen to maximize distance between them. Each inflorescence was monitored for 5 min by one of four observers, during which time all insects interacting with floral reproductive organs were recorded using visual identification and photography (Nikon D3400 digital camera). This time period allowed us to maximize daily site visits and minimize potential bias due to changes in temperature or weather patterns across monitoring events. Because we performed nonlethal visual identification of pollinators, we identified and grouped individuals to the lowest taxonomic level possible in the absence of trapping or morphological measurements (Table A1.2 in Supporting Information).

Several covariates were measured to determine their influence on occupancy and/or detection of pollinators. The site covariate “distance from urban center” was measured as the linear distance (km) from the Virginia State capital building in downtown Richmond, Virginia (data obtained from Google Map 2021; https://maps.google.com). Estimated garden area was determined in person at each site by a visual approximation (meter‐lengths) in the field. Our survey covariates included “garden bloom richness,” “Julian date,” “temperature,” “light level,” and “time of day” (Table 1). Temperature (°C) and light level (lux) were measured using a digital thermometer (Forestry Suppliers digital max/min thermometer) and light meter (Extech Instruments), respectively. Site bloom richness was defined as the number of different plant species in bloom at the site during sampling, excluding our focal species. This was measured on survey days by counting the total number of property‐specific co‐blooming plants; sites on which both plants were monitored separately (Figure 2, white circles) were re‐assessed for bloom richness on each visit. The start time of visual monitoring (hours since midnight) and Julian date of monitoring events were also recorded.

**TABLE 1:** Covariates used in multispecies occupancy models examining the insect pollinator community in Richmond, Virginia, USA, showing covariate descriptions (Covariate), whether it was a site or survey covariate (Type), the terminology used for associated logistic regression coefficients (Notation), the a priori hypothesized direction of effect on occupancy (Expected *ψ*), and the a priori hypothesized direction of effect on detectability (Expected *p*). Note that there is no hypothesized effect on occupancy of survey covariates. Covariates were all normalized by subtracting the mean and dividing by the standard deviation (Standardization Parameters). Site covariates were tested in both occupancy and detection sub‐models, whereas survey covariates were only used in detection.

Covariate	Type	Notation (*ψ*, *p*)	Expected *ψ*	Expected *p*	Standardization parameters (mean, SD)
Dist. to urban center (m)	Site	betalpsi1, betalp6	unc	unc	15,124.1, 12,355.9
Garden area (m^2^)	Site	betalpsi2, betalp7	+	+	464.7, 430.7
Survey start time (hours after midnight)	Survey	betalp1	NA	+	12.9, 1.9
Julian date	Survey	betalp2	NA	unc	193.2, 8.8
Temperature (°C)	Survey	betalp3	NA	+	32.4, 5.0
Light level (lux)	Survey	betalp4	NA	+	42,160.6, 25,340.9
Bloom richness (no. of blooming plant species)	Survey	betalp5	NA	+	19.6, 8.5

*Note:* + = as covariate value increases, probability is hypothesized to increase; NA = covariate is not applicable to parameter; unc = hypothesized direction of effect is uncertain.

### 2.2 Multispecies Occupancy Modeling

We constructed detection histories for all pollinator taxa by recording “1” if a taxon was visually detected during a particular replicate inflorescence observation at a particular site (i.e., garden) and recording “0” otherwise. These data were arranged into an *i* × *j* × *k* array, where *i* is the number of taxa detected at least once, *j* is the number of sites surveyed, and *k* is the maximum number of spatial replicates surveyed at each site. For sites where only one plant species was surveyed, replicates corresponding to the unsurveyed plant were recorded as “NA,” indicating missing observations. Because the two plant species were surveyed at different times within the same summer, our models assume that pollinator occupancy status did not change between the first survey and the last survey (i.e., true occupancy state was constant between surveys). We supplemented these data with 35 all‐zero detection histories for data augmentation, based on pollinator taxa that we did not detect but are known to occur in the region (Ruppel et al. 2019; Ostrom and Grayson 2021). The NA structure of the raw data was propagated into the data augmentation section (e.g., if *L. spicata* was not surveyed at a particular site, then it was also coded as NA for that site in the data augmentation detection histories). Because *L. spicata* and *P. muticum* are in different families, bloom at different times, and have a different floral structure, we expected detection probabilities to vary between the two plant species (at least for some pollinators). Thus, all our models also incorporated a *j* (sites) × *k* (spatial replicates) matrix of binary indicator variables (denoted *ptcov* for plant covariate below) designating the plant species on which a particular observation occurred.

We adapted the MSOM with data augmentation and covariates from Kéry and Royle (2016b) and implemented models via R (v4.3.2; R Core Team 2023) and JAGS (v4.3.1; M. Plummer 2003); see Dryad data repository https://doi.org/10.5061/dryad.nzs7h450q for a full list of accessory packages and software versions used. Our base model was:

$$w_{i}\sim \text{Bernoulli}\left(\Omega \right)$$

$$z_{\mathrm{ij}}|w_{i}\sim \text{Bernoulli}\left(w_{i}\psi_{i}\right)$$

$$y_{\mathrm{ijk}}|z_{\mathrm{ij}}\sim \text{Bernoulli}\left(z_{\mathrm{ij}}p_{\mathrm{ijk}}\right)$$

$$\text{logit}\left(\psi_{i}\right)\sim \text{Normal}\left(\mu_{\text{lpsi}},\sigma_{\text{lpsi}}^{2}\right)$$

$$\text{logit}\left(p_{\mathrm{ijk}}\right)={\mathrm{lp}}_{i}+\left(\text{betalp}.f_{i}\times {\text{ptcov}}_{\mathrm{jk}}\right)$$

$${\mathrm{lp}}_{i}\sim \text{Normal}\left(\mu_{\mathrm{lp}},\sigma_{\mathrm{lp}}^{2}\right)$$

$$\text{betalp}.f_{i}\sim \text{Normal}\left(\mu_{\text{betalp}.f},\sigma_{\text{betalp}.f}^{2}\right)$$

where *w*_*i*_, *z*_*ij*_, and *y*_*ijk*_ are binary indicators of, respectively, whether species *i* is in the regional community (supercommunity process), whether species *i* is present at site *j* (ecological state process), and whether species *i* is detected on replicate survey *k* at site *j* (observation process), respectively. Realizations of these variables are governed by probabilities Ω, *ψ*_*i*_, and *p*_*ijk*_, respectively the probabilities of community membership, site occupancy by species *i* (conditional on community membership), and detection of species *i* at site *k* during replicate *j* (conditional on community membership and site occupancy by the species). We incorporated the plant‐species covariate (*ptcov*_*jk*_) into the detection sub‐model using a logit link, $\text{logit}\left(p_{\mathrm{ijk}}\right)=\ln\left(\frac{p_{\mathrm{ijk}}}{1-p_{\mathrm{ijk}}}\right)$, with *lp*_*i*_ corresponding to a species‐specific intercept indicating detection probability on *P. muticum* , and *betalp.f*_*i*_ corresponding to a species‐specific regression coefficient indicating the change in detection probability on *L. spicata* . *μ*_*lp*_, *σ* ^*2*^_*lp*_, *μ*_*betalp.f*_, and *σ* ^*2*^_*betalp.f*_ are the hyperparameters (means and variances) characterizing the community‐level distributions from which the random effects *lp*_*i*_ and *betalp.f*_*i*_ arise. Thus, our models allowed for unique, species‐specific probabilities of occupancy and detection while still sharing information across species in the estimation of the community‐level hyperparameters.

### 2.3 Covariates and Variable Selection

We examined site and survey covariates as possible predictors of occupancy or detection (Table 1). Values of site covariates could vary by site but were the same across all replicates at a given site and were tested on sub‐models for both occupancy and detection. Values of survey covariates could vary both across sites and between replicates at a given site, and could only be incorporated into the detection sub‐model (MacKenzie et al. 2002). To incorporate covariates, the base model above was extended with additional terms:

$$\text{logit}\left(\psi_{\mathrm{ij}}\right)={\text{lpsi}}_{i}+\left(\text{betalpsi}1_{i}\times \text{psicov}1_{j}\right)\ldots$$

$$\text{logit}\left(p_{\mathrm{ijk}}\right)={\mathrm{lp}}_{i}+\left(\text{betalp}.f_{i}\times {\text{ptcov}}_{\mathrm{jk}}\right)+\left(\text{betalp}1_{i}\times \text{pcov}1_{\mathrm{jk}}\right)\ldots$$

$${\text{lpsi}}_{i}\sim \text{Normal}\left(\mu_{\text{lpsi}},\sigma_{\text{lpsi}}^{2}\right)$$

$$\text{betalpsi}1_{i}\sim \text{Normal}\left(\mu_{\text{betalpsi}1},\sigma_{\text{betalpsi}1}^{2}\right)$$

$$\text{betalp}1_{i}\sim \text{Normal}\left(\mu_{\text{betalp}1},\sigma_{\text{betalp}1}^{2}\right)$$

where “…” in the two logistic regression equations indicates that the models may be extended with additional covariate terms (e.g., *betalpsi2*_*i*_, *betalp2*_*i*_, etc.) and accompanying hyperparameters. As above, this formulation allows for unique, species‐specific responses to particular covariates. All continuous covariates were normalized to have a mean of 0 and standard deviation of 1. We tested covariates for correlation, and covariates with an absolute value of Pearson's *r* ≥ 0.6 were not included in the same model. For missing survey covariates (i.e., because one plant type was not sampled at a particular site), we entered the covariate value as 0 (the normalized mean). Because missing covariates also corresponded to missing observations, the values entered had no impact on the posterior distribution, but some numerical value needed to be entered to avoid an error from JAGS.

For Bayesian hierarchical models, selecting the best model is not straightforward with Bayesian Information Criterion (Schwarz 1978) and Deviance Information Criterion (Spiegelhalter et al. 2002) criticized by various authors (Hooten and Hobbs 2015; Broms et al. 2016), while Watanabe‐Akaike Information Criterion (Watanabe 2013) has not yet been systematically evaluated for MSOMs. With no clear model selection procedure that is both well supported in the literature for application to MSOMs and well suited to our objectives, we instead employed a forward stepwise approach that was more heuristic than procedural (see Appendix S2 for a brief review of Bayesian variable selection and a detailed description of our approach). We considered a covariate supported and influential for a given species if the central 95% credible interval (CrI) for the associated species‐specific regression coefficient did not cross 0, since 0 indicates no effect of the covariate, and tested each covariate individually and retained covariates that were influential for multiple species.

We based inferences on a single final model and present posterior means and CrIs of community‐ and species‐level coefficients. We also present posterior probabilities of species‐level coefficients being positive (i.e., the proportion of posterior samples > 0) even if their CrIs cross 0 (Kéry and Royle 2016a). However, we only present results for one completely undetected taxon because the models are unable to distinguish between them and will estimate identical parameters (within MCMC error) for all undetected taxa (Broms et al. 2016; Kéry and Royle 2016b).

In addition to core model parameters, we also retained posterior samples for several derived parameters. MSOMs implementing data augmentation frequently report the number of species in the supercommunity (the sum of the *w*_*i*_ indicator variables in the model) as a derived parameter estimating regional species richness (i.e., gamma diversity) after accounting for imperfect detection (Dorazio and Royle 2005; Kéry and Royle 2016b). However, the supercommunity is often unclearly defined (Guillera‐Arroita et al. 2019), frequently amounting to a largely theoretical statistical population of sites that are in some way similar to those that were sampled. In such cases, interpretation of the number of species “in the supercommunity” becomes similarly unclear. Additionally, it is possible for species to be in the supercommunity but to not actually occur at any of the sites surveyed (i.e., *w*_*i*_ = 1 but *z*_*ij*_ = 0 for all sites, whereas for any taxon detected, as well as some that may have gone undetected, *w*_*i*_ = 1 and *z*_*ij*_ = 1 for at least one site). For studies like ours in which the focus is on estimating richness at the sites we surveyed, not extrapolating beyond to unsampled sites, this can result in an overestimate of species richness. Guillera‐Arroita et al. (2019) instead advocated reporting the number of species that actually occurred at 1 or more sites (i.e., the sum of species for which *z*_*ij*_ = 1 at least once), which may still include some species that went completely undetected. We report the estimated number of taxa in the supercommunity for consistency with other MSOM studies, but we will focus inferences and discussion on the estimated number of taxa actually occurring at our sites. We also present estimates of the total number of taxa at each site (i.e., alpha diversity), and the total number of sites at which each species occurred (Kéry and Royle 2016b). Because these derived parameters are integer‐valued (number of taxa, number of sites), we present posterior medians rather than posterior means as measures of central tendency.

We used minimally informative priors (Table 2) for community hyperparameters because, to our knowledge, MSOMs have never previously been applied to the insect pollinator community in Richmond, and few other studies have applied them to insect pollinators elsewhere (but, see Mourguiart et al. 2021 [insects/orthopterans]). Community mean intercepts for occupancy and detectability (*μ*_*lpsi*_ and *μ*_*lp*_) were given uniform priors and then were transformed onto the logit scale. However, for Ω we followed Guillera‐Arroita et al. (2019) and instead used a Beta(0.001, 1) prior in order to constrain the estimated total number of taxa in the supercommunity close to the number actually observed, unless the data strongly suggested otherwise, thus reducing the likelihood of unrealistically large estimates of the supercommunity. All other priors were specified directly on the logit scale. Because continuous covariates were all normalized, they were on approximately the same scale, thus we could use the same priors for all such parameters (Broms et al. 2016). The slightly different priors for *σ*_*lp*_ and *σ*_*betalp.f*_ relate to plant type, which is categorical. To select the variances for normal priors and the uniform ranges for the priors of standard deviations, we tested a range of specifications with an empty (all observations NA) dataset and visually inspected density plots of the induced priors (Kéry and Royle 2016a). For intercepts, we selected normal variances that resulted in the flattest distribution from 0 to 1 on the probability scale. For regression coefficients, we selected a uniform range that resulted in a central 95% interval that was as close as possible to, or exceeded, the range −5 to 5 (on the logit scale) (Broms et al. 2016).

**TABLE 2:** Summary of priors for multispecies occupancy models of the insect pollinator community in Richmond, Virginia, USA, describing a group of parameters with shared priors (Description), example parameters as used in the text (Example Terms), examples of the same parameters as used in the BUGS code in the associated data repository (Example BUGS Notation), and the priors used for those parameters (Prior). Note that not all terms were included in all models. The parameters of Beta distributions are the shape parameters (*α* and *β*), of Uniform distributions are the lower and upper bounds, and of Normal distributions are the means and variances.

Description	Example terms	Example BUGS notation	Prior
Hyperparameters			
Probability of supercommunity membership	Ω	omega	$\text{Beta}\left(0.001,1\right)$
Community mean intercept	$\mu_{\text{lpsi}},\mu_{\mathrm{lp}}$	mu.lpsi, mu.betalp_fint	$\text{logit}\left(\text{Uniform}\left(0,1\right)\right)$
Community SD intercept	$\sigma_{\text{lpsi}},\sigma_{\mathrm{lp}}$	sd.lpsi, sd.lp	$\text{Uniform}\left(0,4\right)$
Community mean coefficient	$\mu_{\text{betalp}4},\mu_{\text{betalpsi}1}$	mu.betalp4, mu.betalpsi1	$\text{Normal}\left(0,10\right)$
Community SD coefficient	$\sigma_{\text{betalp}4},\sigma_{\text{betalpsi}1}$	sd.betalp4, sd.betalpsi1	$\text{Uniform}\left(0,4\right)$
Community mean *L. spicata* coefficient	$\mu_{\text{betalp}.f}$	mu.betalp_ls	$\text{Normal}\left(0,10\right)$
Community SD *L. spicata* coefficient	$\sigma_{\text{betalp}.f}$	sd.betalp_ls	$\text{Uniform}\left(0,5\right)$
Species‐specific parameters			
Intercept of species *i*	${\text{lpsi}}_{i},{\mathrm{lp}}_{i}$	lpsi[i], lp[i]	$\text{Normal}\left(\mu_{\text{lpsi}},{\sigma_{\text{lpsi}}}^{2}\right)$
Coefficient of species *i* for covariate	$\text{betalp}4_{i},\text{betalpsi}1_{i}$	betalp4[i], betalpsi1[i]	$\text{Normal}\left(\mu_{\text{betalp}4},{\sigma_{\text{betalp}4}}^{2}\right)$
*L. spicata* coefficient of species *i*	$\text{betalp}.f_{i}$	betalp_f[i, 2]	$\text{Normal}\left(\mu_{\text{betalp}.f},{\sigma_{\text{betalp}.f}}^{2}\right)$

We ran all models with three MCMC chains, for 450,000 iterations and discarded the first 100,000 as burn‐in. We also thinned the chains by retaining only every 10th iteration, solely to make the size of output files more tractable (Link and Eaton 2012), resulting in a total of 105,000 posterior samples. We conservatively considered chains to have converged if the Gelman‐Rubin ($\hat{R}$) statistic for all parameters was < 1.05 (Brooks and Gelman 1998). We also assessed convergence by visual inspection of traceplots and posterior density plots.

We evaluated the fit of our final model via the posterior predictive distribution and Bayesian *p* value (Brooks and Gelman 1998; Broms et al. 2016; Kéry and Royle 2016a), and considered Bayesian *p* values > 0.9 or < 0.1 to indicate unacceptable fit. Various statistics have been suggested for Bayesian *p* values, such as a *χ* ^2^ statistic (Carrillo‐Rubio et al. 2014; Kroll et al. 2014; Tobler et al. 2015) or Freeman–Tukey statistic (Kéry and Royle 2016c; Doser et al. 2022). We followed Broms et al. (2016) and based our Bayesian *p* value on model deviance. We also used graphical methods to visually inspect for lack of fit. See Appendix S3 for additional discussion of model assessment methods, details of our implementation, and diagnostic plots.

## 3 Results

Across all 350 surveys, a total of 14 pollinator taxa were visually detected at least once, with a total of 361 detections (counting spatial replicates; Table A1.2 in Supporting Information). Of those detections, 331 were attributable to just five taxa (*Bombus* spp., *Xylocopa virginica* , *Apis mellifera* , Halictidae, and Vespoidea), with the remaining detections among the other nine taxa. However, the preceding counts include detecting the same taxon at the same site during different replicate surveys, so we had 144 unique detections of a taxon at a given location. *Bombus* spp., *X. virginica* , *A. mellifera* , and Halictidae were detected at 18–35 sites, with all other taxa detected at < 10 sites (and most < 5). Four taxa were detected only on *P. muticum* , four taxa only on *L. spicata* , and six were detected at least once on both plants (Table A1.2 in Supporting Information).

### 3.1 Variable Selection

Only two of our covariates were site covariates suitable for use in the occupancy sub‐model: distance to urban center and garden area. Garden area yielded no taxa for which the coefficient CrIs did not cross 0, so it was dropped from consideration. For distance to urban center, only Halictidae had coefficient CrIs that did not cross 0, so it was also dropped. Similarly, quadratic versions of these terms, or combinations of them, did not have clear predictive value. Additionally, though not above our threshold of 0.6, urban distance and garden area were fairly highly correlated (*r* = 0.54). Thus, our final model used an intercept‐only sub‐model for occupancy, which still allowed occupancy probability to vary by species.

In the detection sub‐model incorporating only plant species, four taxa had coefficients for which the CrIs did not cross 0, supporting the decision to include this term in all models. We then tested the same covariates used for occupancy in the detection sub‐model, none of which were correlated (Table A4 in Supporting Information). Start time influenced the largest number of taxa (Table A5 in Supporting Information). When included as an interaction between plant species and time, this resulted in 17 influential coefficients (four for the plant main effect, 11 for the time main effect, and two for the interaction itself). Coefficients for temperature were similar to those for time, but with wider CrIs, resulting in fewer coefficients reaching the threshold to be considered influential. We dropped non‐influential covariates (CrIs overlapped 0) in the first round including bloom richness, garden area, and interactions between plant and Julian date, light level, and distance to urban center. Quadratic terms generally did not perform well, with only the quadratic for temperature influential for > 1 species. Because the interaction between plant type and survey start time resulted in the largest number of influential coefficients, we selected this as the new base model.

In the second modeling round, no additional main effects were excluded, but interactions between start time and the remaining covariates (light level, distance to urban center, and Julian date) were all excluded due to little support (CrIs overlapped 0). We did not test additional interactions between plant type and the remaining covariates because these were already excluded in the previous round. Distance to urban center was selected as the next base model (psi ~ 1, *p* ~ (plant × start time) + urban distance).

In the third round, the main effect of temperature was only influential for a single species‐specific coefficient, though it had previously been influential for as many as three in simpler models. This indicates that variation in the data potentially explained by temperature was now better explained by other covariates and temperature was excluded from further models. Because temperature's main linear effect was excluded, the quadratic formulation of temperature also was not considered further. An interaction between urban distance and Julian date was influential for two species‐specific coefficients and was retained as the next base model (psi ~ 1, *p* ~ (plant × start time) + (urban distance × Julian date)). In the fourth round, only a few combinations of the remaining variables were possible, so these were attempted and we selected our final model as psi ~ 1, *p* ~ (plant_species × survey start time) + (urban distance × Julian date) + light level (raw data, data preparation R code, and BUGS/JAGS code for the final model, including deviance calculations for Bayesian *p* value available at Dryad: https://doi.org/10.5061/dryad.nzs7h450q).

### 3.2 Model Diagnostics

In our final model, Gelman‐Rubin $\hat{R}$ values for all tracked parameters ranged from 1.001 to 1.049 (1.001–1.021 if parameters related solely to the posterior predictive distribution are excluded), indicating the MCMC chains converged. Similarly, visual inspection of traceplots and posterior density plots revealed no evidence of convergence problems. Most of the primary model parameters converged after 300,000 iterations, but the additional 150,000 iterations were needed to ensure convergence of some parameters related to goodness‐of‐fit. Our Bayesian *p* value was 0.61, indicating acceptable fit though diagnostic plots (Appendix S3, Figures A3.1–A3.3 in Supporting Information) did suggest some minor lack of fit, especially for the most highly detected taxa (Figure A3.3 in Supporting Information). But given the overall symmetry of Figure A3.1 in Supporting Information, and no other evidence of systematic lack of fit, we do not consider these discrepancies severe enough to compromise our community‐level inferences.

The expected deviances from the posterior predictive distribution (3.79–5221.91) were more variable than those from the observed data (1349.43–2142.66) (Figure A3.1 in Supporting Information), but both had similar central tendencies and were relatively evenly distributed above and below a 1:1 reference line. Examining deviance contributions by site from the observed data revealed no major patterns of lack of fit, either across all sites or when subsetting sites by the flower type. There were also no obvious patterns across distance from urban center (Figure A3.2 in Supporting Information). However, deviance contributions by taxon were higher and more variable for our most detected taxa ( *A. mellifera* , Halictidae, *Bombus* spp., *X. virginica* , and, to a lesser extent, Vespoidea), though this also appeared unrelated to plants surveyed (Figure A3.3 in Supporting Information).

### 3.3 Parameter Estimates

#### 3.3.1 Community Hyperparameters

**FIGURE 3:**
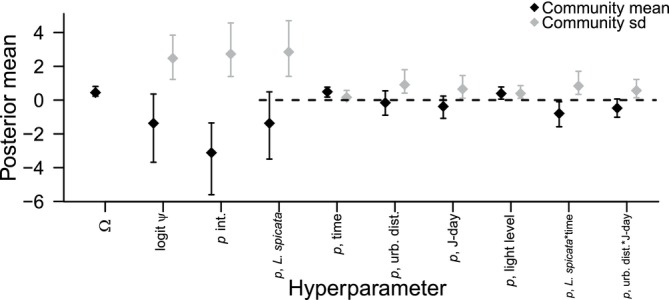
Posterior means and 95% CrIs of community hyperparameters (means in black and standard deviations in gray). Ω is a probability, all others are on the logit scale. The dashed black line is a reference line at 0. Whether or not CrIs cross 0 is not meaningful for intercepts, so no reference line is shown for Ω, logit *ψ*, or *p* intercept. The reference line is also not meaningful for standard deviations (which must be positive), only for means of slope coefficients. All terms except Ω and logit *ψ* pertain to the detection sub‐model. Hyperparameters on the *x*‐axis are: Ω = probability of community membership; logit *ψ* = intercept of the occupancy sub‐model; *p* int. = intercept of the detection sub‐model, corresponding to the main effect of detection on *Pycnanthemum muticum*; *p*, *Liatris spicata* = main effect of detection on *L. spicata*; *p*, time = main effect of time of day; *p*, urb. dist = main effect of distance from urban center on detection; *p*, Jday = main effect of Julian date on detection; *p*, light level = main effect of light level on detection; p, L. spicata*t = interaction between detection on *L. spicata* and time of day; and *p*, urb. dist*J‐day = interaction between distance from urban center and Julian date.

The posterior mean of the probability of membership in the supercommunity (Ω) was 0.45 (95% CrI 0.23–0.81) (Figure 3), compared to 14 taxa that were actually observed out of our data‐augmented data set of 49 possible taxa (proportion = 0.29). The community means of the intercepts for both the occupancy and detection sub‐models were both fairly low at −1.37 (95% CrI −3.68 to 0.36) and −3.12 (−5.60 to −1.35) on the logit scale, respectively (corresponding to 0.20 and 0.04 on the probability scale). However, the corresponding standard deviations were comparatively high (occupancy: 2.47, 95% CrI 1.22–3.84; detection: 2.73, 95% CrI 1.40–4.57). Similarly, the posterior mean for the main effect of plant species was low (−1.37), indicating generally lower detection probability on *L. spicata* than on *P. muticum* , but the 95% CrI crossed 0 (−3.49 to 0.49) and the standard deviation was relatively high (2.85, 95% CrI 1.41–4.70).

Posterior means for the main effect of survey start time and light level were both positive with CrIs that did not cross 0 (time: 0.49, 95% CrI 0.18 to −0.77; light level: 0.39, 95% CrI 0.06–0.78), while the interaction between plant type and start time was negative and also did not cross 0 (−0.79, 95% CrI −1.58 to −0.10). The CrIs for the remaining coefficients all crossed 0.

#### 3.3.2 Species‐Specific Parameters

**FIGURE 4:**
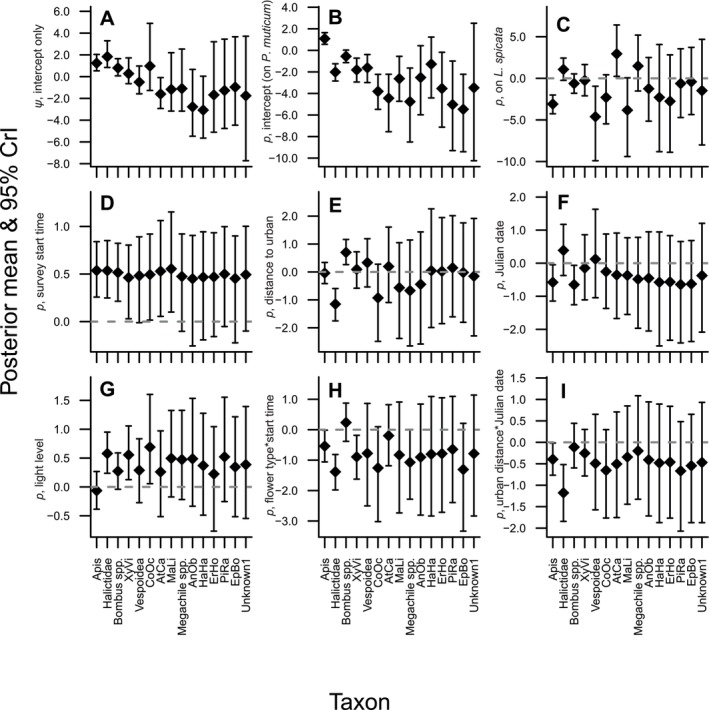
Posterior means and 95% CrIs of species‐specific regression coefficients (logit scale) from selected multispecies occupancy model examining pollinator occupancy and detectability across gardens in the greater Richmond, Virginia, USA region. All parameters except *ψ* are components of the detection (denoted *p*) sub‐model. Shown are occupancy probability (*ψ*, intercept only) (A); intercept of detection probability (corresponding to detectability on *Pycnanthemum muticum*) (B); main effect of detection probability on *Liatris spicata* (C); main effect of survey start time (D); main effect of distance to urban center (E); main effect of Julian date (F); main effect of light level (G); interaction between flower type and start time (H); and interaction between urban distance and Julian date (I). Gray dashed lines at reference lines at 0 (not shown for A or B because they are intercepts). All *x*‐axes are arranged in the same order from taxa with the most detections (left) to the fewest detection (right) and are labeled according to abbreviations in Table A1.2 in Supporting Information.

The 95% CrIs of species‐specific parameters were generally narrowest for the four to five most detected taxa ( *A. mellifera* , Halictidae, *Bombus* spp., *X. virginica* , and Vespoidea; Table A1.2 in Supporting Information), and then became notably less precise as the total number of detections dropped below 15 (Figure 4). Posterior means of species‐specific occupancy probabilities (*ψ*) ranged from −3.08 to 1.84 on the logit scale (0.04–0.86 on the probability scale) (Figure 4A), indicating that our choice of priors still allowed substantial variation in the species‐specific probabilities. Similarly, posterior means of the intercept of the detection sub‐model (corresponding to detectability on *P. muticum* ) ranged from −5.47 to 1.08 (0.004–0.75 on the probability scale) (Figure 4B). The main effects of the change in detectability on *L. spicata* compared to *P. muticum* were highly variable across taxa (Figure 4C). Halictidae (1.07), and *Atalopedes campestris* (2.94) both had positive posterior means on the logit scale, indicating these taxa were more detectable on *L. spicata* . Though *A. campestris* was the only one of these three for which the 95% CrI did not cross 0, for Halictidae the posterior probability of being positive was 0.94, while for *Megachile* spp. it was 0.81 (Table A6 in Supporting Information). The posterior means for all other taxa were at least slightly negative, though several were either close to 0 or had wide 95% CrIs, indicating little evidence that they preferred one plant over the other. Of the taxa with strongly negative coefficients (indicating higher detectability on *P. muticum* ), *A. mellifera* and Vespoidea had 95% CrIs that did not cross 0, while *Coelioxys octodentatus* and *Macrosiagon limbatum* had posterior probabilities of being negative ≥ 0.95.

Among the species‐specific coefficients related to covariates, the posterior means for the main effect of survey start time were all positive (Figure 4D), indicating that all taxa became more detectable later in the day. The 95% CrIs did not cross 0 for 7 taxa and all taxa had posterior probabilities of being positive ≥ 0.94 (Table A6 in Supporting Information). The main effect of distance to urban center (Figure 4E) was negative for Halictidae (higher detections closer to the urban center) and positive for *Bombus* spp. (higher detection farther from the urban center), with CrIs that did not cross 0. Additionally, the posterior probability of *C. octodentatus* being negative was 0.93 (Table A6 in Supporting Information). All other taxa were close to 0 or had wide CrIs for urban distance. *A. mellifera* and *Bombus* spp. were negative for Julian date (higher detection earlier in the year) with CrIs that did not cross 0 (Figure 4F) but CrIs were wide for all other taxa. The posterior means for the main effect of light level (Figure 4G) were all positive except for *A. mellifera* , and CrIs did not cross 0 for Halictidae, *X. virginica* , and *C. octodentatus*. Six other taxa had posterior probabilities of being positive > 0.85.

**FIGURE 5:**
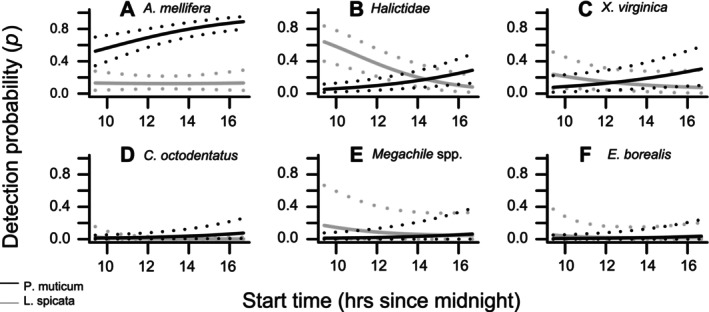
Expected detection probabilities for flower types and time (other covariates held constant at mean values) for taxa with > 90% posterior probability of flower × time interaction coefficient being non‐zero. Plant species indicated by black = *Pycnanthemum muticum* and gray = *Liatris spicata*. Dotted lines are 95% credible intervals. Pollinator taxa are: (A) *Apis mellifera*, (B) Halictidae, (C) *Xylocopa virginica*, (D) *Coelioxys octodentatus* (E) *Megachile* spp., (F) *Epilachna borealis*, which is known as a poor pollinator compared to the others.

**FIGURE 6:**
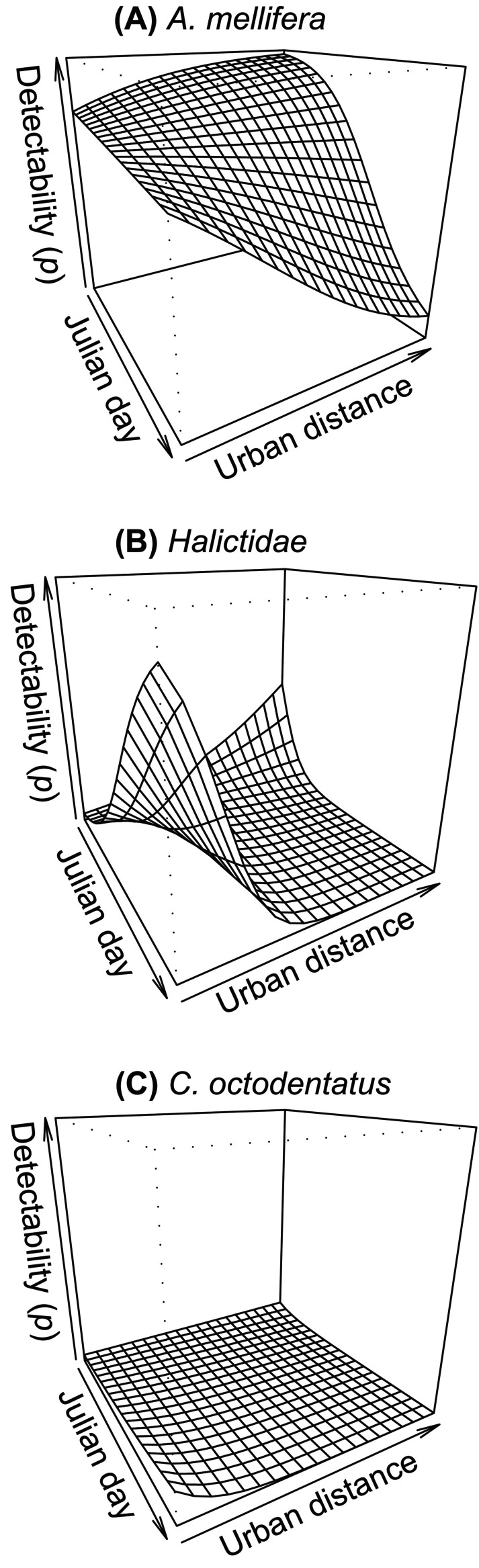
Expected detection probability (*p*) varying by distance from urban center (urban distance) and Julian date for taxa with > 0.9 posterior probability of being non‐zero. All other continuous covariates held constant at mean values and expected detection on plant species *Pycnanthemum muticum*. Pollinator taxa are: (A) *Apis mellifera*, (B) Halictidae, (C) *Coelioxys octodentatus*.

Expected detection probabilities for some taxa were complicated by interactions between plant type and survey start time (Figure 5) and between urban distance and Julian date (Figure 6). For the interaction between plant type and time (Figure 4H), coefficient CrIs did not cross 0 for *A. mellifera* , Halictidae, *X. virginica* , and additionally the posterior probabilities of being negative were > 0.90 for *C. octodentatus*, *Megachile* spp., and *Epilachna borealis* . For *A. mellifera* , expected detection probability on *L. spicata* stayed low (< 0.30) and fairly constant across the survey times, but detectability on *P. muticum* was predicted to be consistently higher (> 0.30) and to increase over the course of the day (Figure 5A). Expected detectability of Halictidae was high on *L. spicata* earlier in the day (~0.60), but dropped off notably as the day progressed, while detectability on *P. muticum* was low (~0.05) and increased later in the day, albeit more gradually and with substantial overlap of CrIs in the afternoon (Figure 5B). The expected detectability of *X. virginica* exhibited a similar pattern to Halictidae, but with a less pronounced difference between plants in the morning and more overlap of CrIs throughout the day (Figure 5C). For the remaining three taxa (Figure 5D–F), expected detectability was low on both plants, with wide overlapping CrIs.

For the interaction between urban distance and Julian date, only *A. mellifera* and Halictidae had CrIs that did not cross 0, though the posterior probability of *C. octodentatus* being negative was 0.92 (Figure 4I). Expected detection of *A. mellifera* was highest early in the season far from the urban center, and lowest late in the season far from the urban center (Figure 6A). Late in the season, as distance to urban decreased, the detectability of *A. mellifera* , increased, but early in the season closeness to urban resulted in a slight decrease in detection. Thus, as the season progressed, detectability generally declined, but it declined much more sharply farther away from the urban center than close to it. For Halictidae, detectability was also lowest late in the season and far from the urban center but it was also predicted to be nearly as low early in the season close to the urban center (Figure 6B). Expected detectability was highest late in the season close to the urban center but had another strong peak early in the season far from the urban center. As the season progressed, detectability was initially highest far from the urban center before briefly becoming low across all distances, then increasing closer to the urban center. Expected detectability of *C. octodentatus* was slightly higher late in the season close to the urban center, but was consistently low across distances and the season, showing little evidence of a meaningful effect (Figure 6C).

#### 3.3.3 Derived Parameters

**FIGURE 7:**
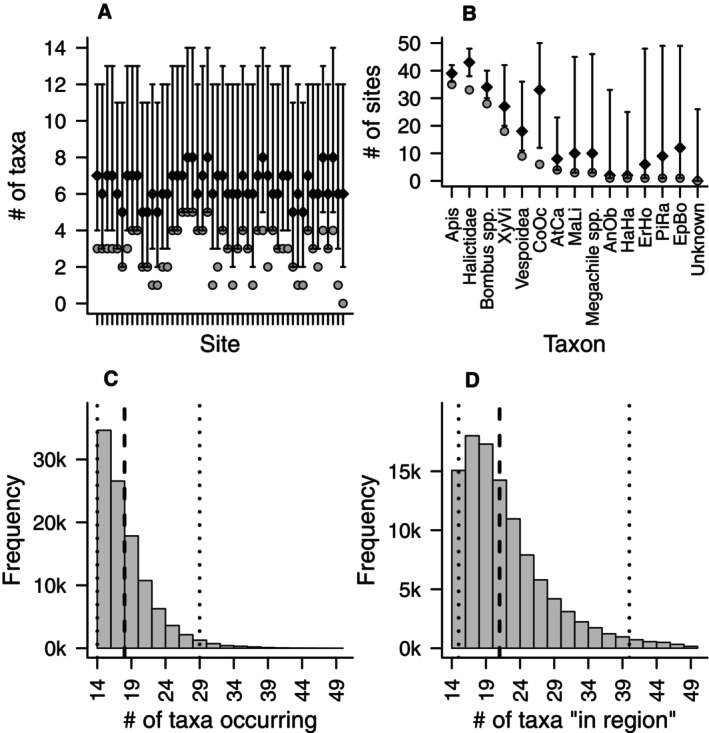
Derived parameters for pollinators in Richmond, Virginia. Top figures, black diamonds are posterior medians, gray circles are observed counts (or naïve occupancy for B), error bars are 95% CrIs, # = number. Bottom figures, dashed lines are posterior medians, dotted lines are central 95% CrIs. Shown are the number of taxa estimated to be at each site (A); number of sites at which each taxon is estimated to occur (left axis) or species‐specific occupancy probability (right axis) (B); the number of taxa estimated to occur in at least 1 site (including those which may have gone completely undetected) (C); and the number of taxa estimated to be present “in the community” (though some may not have been present at any of the sites surveyed) (D).

The posterior medians of the number of taxa per site, after accounting for imperfect detection, ranged from 5 to 8, with fairly wide overlapping CrIs, showing little evidence of differences in richness across sites (Figure 7A). Estimated richness was consistently higher than the number of taxa actually observed, suggesting that in many cases, roughly half of the taxa that were actually present went undetected. Posterior medians of the number of sites at which each taxon is expected to occur ranged from 2 to 43 (0–43 if including a representative undetected taxon), with a handful of widely distributed taxa and the remainder expected to occur at relatively few sites (Figure 7B). *A. mellifera* , Halictidae, *Bombus* spp., and *C. octodentatus* were expected to occur at 33 or more of the 50 sites; *X. virginica* was expected to occur at 27; Vespoidea and *E. borealis* at 12–18; and the remaining seven taxa at 10 or fewer sites. However, CrIs were fairly wide except for our three most detected taxa ( *A. mellifera* , Halictidae, and *Bombus* spp.), indicating substantial uncertainty in many estimates.

The posterior median of the number of taxa occurring at all sampled sites (Figure 7C) was 18 (95% CrI 14–29), indicating that around a quarter of the taxa present at our study sites went completely undetected. By comparison, the posterior median of the number of taxa in the supercommunity (Figure 7D) was 21 (95% CrI 15–40), with the additional taxa not actually occurring at any site. Uncertainty in the latter parameter was also larger with the CrI spanning most of the range of the data‐augmented taxa. The center of posterior mass was well to the left of the data‐augmentation maximum and well within the range of plausibility, indicating that the data augmentation was sufficient to avoid truncation (Kéry and Royle 2016b) and that the model was not attempting to estimate an unrealistically large number of taxa (Guillera‐Arroita et al. 2019).

## 4 Discussion

### 4.1 Study Goals and Key Findings

Our goal was to characterize and estimate the magnitude of predictors of pollinator occupancy and detection probabilities at Mid‐Atlantic region (USA) urban and suburban sites. Additionally, we determined pollinator diversity and species‐specific occupancy across sites using MSOM modeling. As our study sites were predominantly on private land with landowners who actively cultivated *L. spicata* and/or *P. muticum* , we expected to draw pollinators despite site‐specific variation in land use (e.g., garden area and distance to urban center) (Table 1). We found no covariates that influenced occupancy, but several covariates influenced detection for multiple species.

We estimated (posterior medians) 5–8 taxa per site with similar pollinator richness (alpha diversity) across sampled locations. Similarity in diversity at each site is unsurprising considering we did not find support for any occupancy covariates. Across all sampled sites in the landscape (gamma diversity), 18 total taxa (95% CrI 14–29) were expected to occur, 4 of which went entirely undetected during surveys. The diversity values, which are lower than expected, reflect our more cautious approach to taxa identification and classification (i.e., genus‐level identifications in some cases due to noninvasive, visual monitoring survey techniques). For example, we classified all bumblebees collectively as *Bombus* spp. even though several species are known locally. Ostrom and Grayson (2021) reported the trapping of three different *Bombus* spp. in their Richmond‐area survey, and a more recent survey by our group detected six different species visiting *P. muticum* specifically (Ortiz et al. 2024). These *Bombus* species are typically polylectic and capable of traveling long distances, traits that may contribute to their detectability and broader presence across the landscape (Mola and Williams 2025).

The taxa that make up the core community and were most commonly predicted occupants across sites included *A. mellifera* , Halictidae bees, *Bombus* spp., and *X. virginica* (eastern carpenter bee), while other taxa were estimated to be more localized or had high uncertainty in estimates. Our models suggested that approximately half of the taxa expected to occur at a given site were actually detected. This underscores the importance of analyses that account for imperfect detection when surveying for pollinators. All survey techniques have some bias that may influence detection (Hutchinson et al. 2022), but the benefit of visual monitoring is that it anchors pollination events to a specific flower and time, allowing for direct tallying of plant–pollinator interactions. However, this technique can be a laborious, time‐intensive process that may also be confounded by survey‐specific variables (e.g., weather events). In contrast, pollinator surveys using lethal traps, collect a wide variety of insects, providing a broader inventory of local extant species that are often undetected during visual surveys, but are unable to directly assess specific plant–insect interactions (Hutchinson et al. 2022). In the future, a combination of both approaches may lead to stronger inferences.

### 4.2 Urban Pollinator Communities and Species‐Specific Occupancy

The presence of taxa we found concurred with other recently described regional pollinator studies (Ruppel et al. 2019; Kammerer et al. 2020; Ostrom and Grayson 2021). Two of these studies (Kammerer et al. 2020, Ostrom and Grayson 2021) reported several additional bee taxa that went undetected in our survey; however, their methodology used trapping techniques adjacent to sites with general floral resources (i.e., not *L. spicata* or *P. muticum* ) instead of direct visual observations. A recent pollinator study using malaise traps identified predominantly non‐syrphid Diptera, with bees representing only 6% of their collection (Ganuza et al. 2022). The predominance of bee taxa at most sites in our survey highlights the need to monitor pollinator populations using a variety of methodologies.

It can be tempting to interpret hyperparameters, especially the community means, analogously to regression coefficients, for example, a negative estimate with a CrI that does not cross 0 represents a negative effect of the associated covariate. However, it is important to remember that these are random effects, and thus constitute somewhat informative priors (informed by the other taxa in the analysis) on the species‐specific estimates (Kéry and Royle 2016b). Thus, the community mean hyperparameter for a covariate does not necessarily represent the average effect across the detected taxa, but rather the average of a hypothetical statistical population of which those taxa are a sample. As with other priors, taxa for which sample size is relatively large may result in posterior estimates that differ from the prior, while taxa with smaller sample sizes will have posterior estimates that tend towards the community mean. This “Bayesian shrinkage” is usually discussed as one of the key advantages of the MSOM framework (Broms et al. 2016) because it allows stronger inferences about rarely detected taxa, at the expense of minor bias towards the community mean. Thus, a community mean with a strong effect size could indicate a notable overall pattern in the community, but there might still be individual taxa with a substantially different effect size and direction of such effect, especially if the associated standard deviation is high. For example, some covariates could affect multiple taxa strongly, but in opposite directions for some, in which case the community mean might be very close to 0, even though the covariate was important for specific taxa.

We did not find support for community‐level effects on occupancy, which is noteworthy as our models suggest that pollinator occupancy across taxa was not affected by the scale of urbanization across sites (i.e., distance to urban center). Amid the ongoing expansion of human populations and development, others have attempted to evaluate the extent to which urbanization impacts the presence and diversity of insects, including pollinators. These studies have ranged in scale from genus‐level evaluations to the broader arthropod community, and their outcomes have been widely variable. For example, Boone et al. (2023) found that several bumblebee species in their survey, including the endangered *Bombus affinis* , were more likely to occupy developed areas compared to agricultural or seminatural areas, possibly due to the availability of nesting habitat (Boone et al. 2022). Other bumblebees (e.g., *B. borealis* and *B. ternarius* ) in their survey, however, were more likely to occupy seminatural settings. Similar to our findings, MacIvor and Packer (2016) reported no correlation between urbanization and the occupancy of numerous solitary bee species, when defining urbanization by the proximity to surrounding buildings. Others, when assessing the broader arthropod community, have reported limited effects due to distance from seminatural areas for most families under observation (Lewthwaite et al. 2024). Some studies, however, described a negative impact on pollinator occupancy due to the extent of nearby impervious surfaces, which can be used as a metric for “urbanness” (Berthon et al. 2021). Collectively, these studies demonstrate the challenges of modeling pollinator (and other arthropod) occupancy in the urban setting, as the unique collective of local covariates makes it difficult to compare across studies. They do, however, suggest that the urban environment, especially areas supplemented with ample space and floral diversity, can be supportive of pollinator communities (Baldock et al. 2019; Ganuza et al. 2022).

### 4.3 Ecological Meaning and Management Implications

Detection covariates are sometimes characterized as nuisance parameters that must be accounted for to reduce bias in occupancy estimates but are of little biological interest. However, information about detection parameters can improve future sampling efforts and suggest avenues for future research. This is especially true of community studies in which the goal is to optimize effort to maximize detections of multiple species. The largest community effect size on detection we found was regarding the plant species surveyed ( *P. muticum* or *L. spicata* ) (posterior mean −1.37, lower on *L. spicata* ), but the associated standard deviation was also high (posterior mean 2.85), indicating substantial variation among insect taxa. The negative estimate is likely due to more detections of taxa that were only seen on *P. muticum* than those only seen on *L. spicata* (23 vs. 13 detections) and because *A. mellifera* was detected far more often on *P. muticum* (101 vs. 22 detections on *L. spicata* ), possibly due to the proximity of *P. muticum* sites to honey bee hives. Nevertheless, several taxa were expected to have higher detection probabilities on *L. spicata* , or to exhibit little preference between the plant types (see species‐specific estimates below).

Other detection hyperparameters were close to 0 with narrower CrIs and lower standard deviations, indicating more consistency across the insect community in response to these covariates, but still allowed species‐specific variation, especially for frequently detected species. Community means for the main effect of survey start time and light level were slightly positive indicating generally higher detectability later in the day (surveys were conducted from roughly 9:20 am to 4:45 pm) and at higher light levels. Later times of day generally corresponded to higher temperatures, which could lead to increased insect activity (Ruppel et al. 2019). Interestingly though, during our model selection process we did not find support for temperature as a covariate. We attribute this to the fact that time of day was recorded for each replicate survey (up to 10 times per site) whereas temperature was only measured once per site visit (no more than twice per site). Thus time of day was a more information‐rich variable and reflected change over the course of a single day, even though we suspect temperature was the ultimate cause. Light levels were also measured for each replicate survey and might be expected to similarly mirror time of day and temperature, but correlations were low between light level and time of day and between light level and temperature. Light levels may instead have been primarily affected by canopy cover and/or cloud cover and other weather conditions.

Community mean hyperparameters were slightly negative for the interactions of plant type with time of day and for urban distance with Julian date, and again the associated standard deviations were relatively low. This supports the inclusion of the interactions in the model, despite the added complexity. When species‐specific estimates of interactions for a given taxon were not statistically different than 0, the interaction effectively drops out and estimated detectability was driven by the main effects only. The community means for the main effects of urban distance and Julian date were close to 0, either because of strong species‐specific effects but with opposite signs, or because the covariate was only influential for a few species, with estimates for the majority close to 0.

For species‐specific detection parameters, we focus discussion on “well‐supported” estimates, those with > 90% posterior probability of being non‐zero (Table A6 in Supporting Information) in order to include not only clearly supported estimates (95% CrI does not cross 0) but also strongly suggestive estimates (CrI crosses 0 only slightly). Thus, species‐specific estimates that more widely crossed 0 were not considered statistically different from 0 and would have little impact on detection probability estimates for that taxon. Six taxa had well‐supported estimates for the main effect of plant type, four of which indicated a preference for *P. muticum* (*A. mellifera*, Vespoidea, *C. octodentatus*, and *M. limbatum*), and two a preference for *L. spicata* (Halictidae and *A. campestris* ) despite the fact that *P. muticum* was more widely available across sites. Six of those ( *A. mellifera* , Halictidae, *C. octodentatus, X. virginica*, *Megachile* spp., and *E. borealis* ) also had well‐supported estimates of the interaction between plant type and time of day. While the interactions are complex and warrant further species‐specific investigation, these results broadly reinforce the decision to conduct surveys on multiple species of native plants. Had surveys been limited to a single plant species, we likely would have missed even more insect taxa that were in fact present. Future surveys could expand our approach to include more than two native plants with different structural or phenological characteristics that might be preferred by taxa for which we had small sample sizes (or missed completely), which would also improve sample sizes. In the Mid‐Atlantic, Johnson et al. (2017) noted numerous potential high‐quality native plant candidates for evaluation including several *Rudbeckia* and *Solidago* species. Also, several features of plants surveyed may have led to our observations and can be used when considering conservation efforts for low‐abundance species with similar known functional characteristics. *P. muticum* may provide a higher quantity of nectar and pollen per unit area despite its smaller individual flower size (Weakley et al. 2012). The nectar and pollen may also be more nutritious and/or accessible, which could lead to a wider array of pollinators and other insects competing for such resources. It is likewise possible that *Pycnanthemum* spp. bloom seasonality coincides with seasonal emergence of a mostly unique, more diverse pollinator population, or that they invest more heavily in pollinator attraction during their long bloom period (Setzer et al. 2021). Plants in this genus are known to produce volatiles that could influence pollinator attraction (Lawson et al. 2021) or promote some other beneficial element (e.g., health) (Schmitt et al. 2021). *L. spicata* is native to eastern and central North America, preferring moist meadows and other similar habitat (Weakley et al. 2012). Its small flower size and reduced nectar concentration (Arnold and Michaels 2017) are predicted to attract a more limited, and possibly unique, pollinator profile, especially given its prevalence in resource‐poor habitats such as serpentine grasslands (Richins 2020). Johnson et al. (2017) ascribed *L. spicata* a high “species impact index” score for the Mid‐Atlantic region due to its potential to positively impact urban pollinator populations and remnant wild native plants.

An additional consideration is that foraging behavior often differs between generalists and specialists, with important ecological implications (Rasmussen et al. 2020). Specialist bees are typically restricted to foraging on a narrow range of floral hosts, sometimes limited to a single plant family or genus, due to such factors as pollen and/or nectar chemistry (Vanderplanck et al. 2017). In contrast, generalist pollinators can forage across a broad range of floral resources and are often more flexible in their diet at broader spatial or temporal scales, even if locally they may exhibit narrower preferences. In our study system, using *P. muticum* (Lamiaceae) and *L. spicata* (Asteraceae), these patterns may have shaped pollinator visitation: while *L. spicata* belongs to a plant family with many locally co‐occurring species (i.e., in urban and suburban green spaces), *P. muticum* is relatively isolated in floral composition, with comparatively fewer other Lamiaceae family members present. As a result, generalist pollinators might more readily shift among *L. spicata* and other Asteraceae species, while specialists may show stronger fidelity or be absent altogether depending on floral availability and compatibility.

We found that detectability was expected to increase for all taxa when our measurements were completed later in the day and for most taxa during brighter conditions. While this could be a case of a few, frequently detected taxa pulling the rest toward the community mean, it is also entirely plausible that exothermic insects become more active (and thus be more detectable) as temperature increases. Again, this suggests ways in which future survey efforts could be improved. Concentrating survey efforts later in the day could maximize detection probabilities and improve sample sizes, and importantly, these within‐day variations should be accounted for in analyses. Measuring temperature for every replicate survey would probably also provide better explanatory power and would facilitate the use of a directly explanatory covariate, rather than a proxy (as we believe time of day is for temperature in this case). Controlling natural outdoor light levels is not feasible, but our results indicate that light level also should be accounted for in analyses.

### 4.4 Modeling Considerations and Future Improvments

Our coarse taxonomic resolution may have resulted in difficulties in drawing ecological conclusions for occupancy for some species, but further subdivision was not possible due to low sample sizes. Nonetheless, we did find well‐supported evidence for its effect on detection for three taxa (two negatively and one positively), two of which also had well‐supported estimates for the interaction between urban distance and Julian date. Julian date was well‐supported for two taxa (both negatively, meaning higher detection earlier in the season), one of which also had a well‐supported interaction term (3 total interactions). Of the four taxa with at least one of these terms well‐supported, three of them were our three most detected taxa ( *A. mellifera* , Halictidae, and *Bombus* spp.). For the remaining 10 taxa, one was detected 28 times, one 15 times, and the remainder < 8 times each, and thus it is unclear whether they were genuinely unaffected by urban distance or Julian date, or if sample sizes were simply too small for effects to be discernible. That we did find effects well‐supported for some taxa indicates that potentially, further investigation is warranted for others. For example, the non‐native, managed *A. mellifera* was expected to be detected earlier in the season at rural sites. In North America, *A. mellifera* is commonly observed in rural settings for agricultural pollination services, which is essential for a large number of crops (Klein et al. 2018). In our study, *A. mellifera* detection in a more rural setting, with high detection early in the monitoring period, but transitioning to low detection later, followed a similar trend as reported by others (Danner et al. 2017). Danner et al. (2017) suggested these dynamics likely reflected seasonal colony development characteristics, with honey bees increasing their late season foraging distance and diversifying their food sources. That *A. mellifera* had a lower drop off in urban versus rural detection probability may reflect its generalist pollinator behavior (Goulson 2003) and the increasing urban beekeeping movement, especially in the USA (Sponsler and Bratman 2021).

One way in which urban–rural gradient (urban distance) could affect pollinators that was not explored in our current analysis, is local abundance. Occupancy modeling is based on detection/non‐detection of a species, and basic occupancy models do not distinguish between sites with relatively high or relatively low local abundance, so long as the species is present. However, in many cases it is reasonable to believe that at sites with higher local abundance, the probability of detecting at least one member of the species will also be higher (Dorazio et al. 2006; Royle and Dorazio 2009). Royle and Nichols (2003) developed an extension of single‐species occupancy models that incorporates heterogeneity in detection probabilities due to (unobserved) local abundance, and Dorazio and Royle (2005) used a similar approach with MSOMs. We did not consider such an approach because we did not consider our sample sizes sufficient to fit such a model, but it could be an avenue for future exploration with larger sample sizes. Absent such a model, if local abundances varied with distance from urban center, it could produce a pattern similar to what we found in which urban distance influenced detection, at least for some taxa.

## 5 Conclusions

This study demonstrates that MSOMs can be an effective tool for investigating and monitoring the pollinator community. We were able to estimate occupancy probabilities for 14 observed insect taxa, nine of which were detected fewer than eight times, far too few detections to fit a single‐species occupancy model. Uncertainty was admittedly greater and CrIs wider for these rarely detected taxa, but this nevertheless provides a baseline against which future pollinator studies in the greater Richmond, Virginia area can be compared. We were also able to estimate that an additional four (95% CrI 0–15) unknown taxa were present at one or more of our study sites, but went completely undetected. Finally, we were able to estimate the effects of detection covariates that impacted multiple taxa and could provide insight into ways to improve and standardize future pollinator monitoring efforts. Most of those covariates were not well‐supported for taxa with only a few detections, but that is unsurprising given the small sample sizes and corresponding lack of statistical power. The insights that we were able to gain will aid in increasing future sample sizes, leading to stronger inference and new insights.

## Author Contributions

**Nicholas J. Ruppel:** conceptualization (equal), data curation (supporting), formal analysis (supporting), funding acquisition (supporting), investigation (equal), methodology (equal), project administration (supporting), writing – original draft (equal), writing – review and editing (equal). **Robert B. Nipko:** conceptualization (equal), data curation (lead), formal analysis (equal), investigation (equal), methodology (equal), software (lead), writing – original draft (equal), writing – review and editing (equal). **Mackenzie Dingus:** data curation (supporting), investigation (supporting), writing – review and editing (supporting). **Allison C. Ortiz:** data curation (supporting), investigation (supporting), writing – review and editing (supporting). **Teresa Weir:** data curation (supporting), investigation (supporting), writing – review and editing (supporting). **Marcella J. Kelly:** conceptualization (equal), formal analysis (supporting), funding acquisition (supporting), methodology (supporting), software (supporting), writing – review and editing (equal). **Stephanie S. Coster:** conceptualization (equal), data curation (equal), formal analysis (supporting), funding acquisition (lead), investigation (equal), methodology (equal), project administration (lead), writing – review and editing (supporting).

## Conflicts of Interest

The authors declare no conflicts of interest.

## Supporting information


**Appendices S1–S6:** ece372502‐sup‐0001‐AppendicesS1‐S6.zip.

## Data Availability

Data and modeling information supporting this study are archived in Dryad Digital Repository: https://doi.org/10.5061/dryad.nzs7h450q.

## References

[ece372502-bib-0001] Ahrné, K. , J. Bengtsson , and T. Elmqvist . 2009. “Bumble Bees (*Bombus* spp) Along a Gradient of Increasing Urbanization.” PLoS One 4: e5574. 19440367 10.1371/journal.pone.0005574 PMC2679196

[ece372502-bib-0002] Armbruster, W. S. 2017. “The Specialization Continuum in Pollination Systems: Diversity of Concepts and Implications for Ecology, Evolution and Conservation.” Functional Ecology 31, no. 1: 88–100.

[ece372502-bib-0003] Arnold, P. M. , and H. J. Michaels . 2017. “Nectar Sampling for Prairie and Oak Savanna Butterfly Restoration.” Applications in Plant Sciences 5: 1600148. 10.3732/apps.1600148 PMC5499304 28690931

[ece372502-bib-0004] Ayers, A. C. , and S. M. Rehan . 2021. “Supporting Bees in Cities: How Bees Are Influenced by Local and Landscape Features.” Insects 12: 128. 33540577 10.3390/insects12020128 PMC7912800

[ece372502-bib-0006] Baldock, K. C. R. , M. A. Goddard , D. M. Hicks , et al. 2015. “Where Is the UK's Pollinator Biodiversity? The Importance of Urban Areas for Flower‐Visiting Insects.” Proceedings of the Royal Society B: Biological Sciences 282: 20142849. 10.1098/rspb.2014.2849 PMC4345454 25673686

[ece372502-bib-0005] Baldock, K. C. R. , M. A. Goddard , D. M. Hicks , et al. 2019. “A Systems Approach Reveals Urban Pollinator Hotspots and Conservation Opportunities.” Nature Ecology & Evolution 3: 363–373. 30643247 10.1038/s41559-018-0769-y PMC6445365

[ece372502-bib-0007] Bartomeus, I. , M. G. Park , J. Gibbs , B. N. Danforth , A. N. Lakso , and R. Winfree . 2013. “Biodiversity Ensures Plant–Pollinator Phenological Synchrony Against Climate Change.” Ecology Letters 16: 1331–1338. 23968538 10.1111/ele.12170

[ece372502-bib-0008] Bates, A. J. , J. P. Sadler , A. J. Fairbrass , S. J. Falk , J. D. Hale , and T. J. Matthews . 2011. “Changing Bee and Hoverfly Pollinator Assemblages Along an Urban–Rural Gradient.” PLoS One 6: e23459. 21858128 10.1371/journal.pone.0023459 PMC3155562

[ece372502-bib-0009] Berthon, K. , S. T. Meyer , F. Thomas , A. Frank , W. W. Weisser , and S. Bekessy . 2021. “Small‐Scale Habitat Conditions Are More Important Than Site Context for Influencing Pollinator Visitation.” Frontiers in Ecology and Evolution 9: 703311.

[ece372502-bib-0010] Boone, M. L. , E. Evans , A. Wolf , H. Minser , J. Watson , and T. A. Smith . 2022. “Notes From the Rusty Patched Bumble Bee (*Bombus affinis* Cresson) Nest Observations.” Insect Conservation and Diversity 15, no. 3: 380–384.

[ece372502-bib-0011] Boone, M. L. , Z. M. Portman , I. Lane , and S. Rao . 2023. “Occupancy of *Bombus affinis* (Hymenoptera: Apidae) in Minnesota Is Highest in Developed Areas When Standardized Surveys Are Employed.” Environmental Entomology 52: 918–938. 37681665 10.1093/ee/nvad088

[ece372502-bib-0012] Broms, K. M. , M. B. Hooten , and R. M. Fitzpatrick . 2016. “Model Selection and Assessment for Multi‐Species Occupancy Models.” Ecology 97: 1759–1770. 27859174 10.1890/15-1471.1

[ece372502-bib-0014] Brooks, S. P. , and A. Gelman . 1998. “General Methods for Monitoring Convergence of Iterative Simulations.” Journal of Computational and Graphical Statistics 7: 434–455.

[ece372502-bib-0015] Brosi, B. J. 2016. “Pollinator Specialization: From the Individual to the Community.” New Phytologist 210, no. 4: 1190–1194. 27038018 10.1111/nph.13951

[ece372502-bib-0017] Cariveau, D. P. , and R. Winfree . 2015. “Causes of Variation in Wild Bee Responses to Anthropogenic Drivers.” Current Opinion in Insect Science 10: 104–109. 29587998 10.1016/j.cois.2015.05.004

[ece372502-bib-0018] Carrillo‐Rubio, E. , M. Kéry , S. J. Morreale , et al. 2014. “Use of Multispecies Occupancy Models to Evaluate the Response of Bird Communities to Forest Degradation Associated With Logging: Bird Communities and Forest Degradation.” Conservation Biology 28: 1034–1044. 24628427 10.1111/cobi.12261

[ece372502-bib-0019] Danner, N. , A. Keller , S. Härtel , and I. Steffan‐Dewenter . 2017. “Honey Bee Foraging Ecology: Season but Not Landscape Diversity Shapes the Amount and Diversity of Collected Pollen.” PLoS One 12: e0183716. 28854210 10.1371/journal.pone.0183716 PMC5576699

[ece372502-bib-0020] Deguines, N. , R. Julliard , M. de Flores , and C. Fontaine . 2012. “The Whereabouts of Flower Visitors: Contrasting Land‐Use Preferences Revealed by a Country‐Wide Survey Based on Citizen Science.” PLoS One 7: e45822. 23029262 10.1371/journal.pone.0045822 PMC3446938

[ece372502-bib-0021] Dorazio, R. M. , and J. A. Royle . 2005. “Estimating Size and Composition of Biological Communities by Modeling the Occurrence of Species.” Journal of the American Statistical Association 100: 389–398.

[ece372502-bib-0022] Dorazio, R. M. , J. A. Royle , B. Söderström , and A. Glimskär . 2006. “Estimating Species Richness and Accumulation by Modeling Species Occurrence and Detectability.” Ecology 87: 842–854. 16676528 10.1890/0012-9658(2006)87[842:esraab]2.0.co;2

[ece372502-bib-0023] Doser, J. W. , A. O. Finley , M. Kéry , and E. F. Zipkin . 2022. “ spoccupancy: An R Package for Single‐Species, Multi‐Species, and Integrated Spatial Occupancy Models.” Methods in Ecology and Evolution 13: 1670–1678.

[ece372502-bib-0024] Emer, C. , J. Memmott , I. P. Vaughan , D. Montoya , and J. M. Tylianakis . 2016. “Species Roles in Plant–Pollinator Communities Are Conserved Across Native and Alien Ranges.” Diversity and Distributions 22: 841–852.

[ece372502-bib-0025] Fetridge, E. D. , J. S. Ascher , and G. A. Langellotto . 2008. “The Bee Fauna of Residential Gardens in a Suburb of New York City (Hymenoptera: Apoidea).” Annals of the Entomological Society of America 101: 1067–1077.

[ece372502-bib-0026] Frankie, G. W. , R. W. Thorp , M. H. Schindler , B. Ertter , and M. Przybylski . 2002. “Bees in Berkeley?” Fremontia 30: 50–58.

[ece372502-bib-0027] Fuccillo Battle, K. , C. E. De Rivera , and M. B. Cruzan . 2021. “The Role of Functional Diversity and Facilitation in Small‐Scale Pollinator Habitat.” Ecological Applications 31: e02355. 33870597 10.1002/eap.2355

[ece372502-bib-0028] Gallagher, M. K. , and D. R. Campbell . 2020. “Pollinator Visitation Rate and Effectiveness Vary With Flowering Phenology.” American Journal of Botany 107: 445–455. 32086803 10.1002/ajb2.1439

[ece372502-bib-0029] Ganuza, C. , S. Redlich , J. Uhler , et al. 2022. “Interactive Effects of Climate and Land Use on Pollinator Diversity Differ Among Taxa and Scales.” Science Advances 8: eabm9359. 35544641 10.1126/sciadv.abm9359 PMC9075793

[ece372502-bib-0032] Goulson, D. 2003. “Effects of Introduced Bees on Native Ecosystems.” Annual Review of Ecology, Evolution, and Systematics 34: 1–26.

[ece372502-bib-0033] Goulson, D. , E. Nicholls , C. Botias , and E. L. Rotheray . 2015. “Bee Declines Driven by Combined Stress From Parasites, Pesticides, and Lack of Flowers.” Science 347, no. 6229: 1255957. 25721506 10.1126/science.1255957

[ece372502-bib-0034] Guillera‐Arroita, G. , M. Kéry , and J. J. Lahoz‐Monfort . 2019. “Inferring Species Richness Using Multispecies Occupancy Modeling: Estimation Performance and Interpretation.” Ecology and Evolution 9: 780–792. 30766668 10.1002/ece3.4821 PMC6362448

[ece372502-bib-0035] Hall, D. M. , G. R. Camilo , R. K. Tonietto , et al. 2017. “The City as a Refuge for Insect Pollinators.” Conservation Biology 31: 24–29. 27624925 10.1111/cobi.12840

[ece372502-bib-0036] Hanley, M. E. , A. J. Awbi , and M. Franco . 2014. “Going Native? Flower Use by Bumblebees in English Urban Gardens.” Annals of Botany 113: 799–806. 24647914 10.1093/aob/mcu006 PMC3962246

[ece372502-bib-0037] Hennig, E. I. , and J. Ghazoul . 2012. “Pollinating Animals in the Urban Environment.” Urban Ecosystems 15: 149–166.

[ece372502-bib-0038] Hooten, M. B. , and N. T. Hobbs . 2015. “A Guide to Bayesian Model Selection for Ecologists.” Ecological Monographs 85: 3–28.

[ece372502-bib-0039] Hutchinson, L. A. , T. H. Oliver , T. D. Breeze , et al. 2022. “Inventorying and Monitoring Crop Pollinating Bees: Evaluating the Effectiveness of Common Sampling Methods.” Insect Conservation and Diversity 15: 299–311.

[ece372502-bib-0040] Johnson, A. L. , A. M. Fetters , and T. Ashman . 2017. “Considering the Unintentional Consequences of Pollinator Gardens for Urban Native Plants: Is the Road to Extinction Paved With Good Intentions?” New Phytologist 215: 1298–1305. 28626951 10.1111/nph.14656

[ece372502-bib-0041] Kaluza, B. F. , H. Wallace , T. A. Heard , A. Klein , and S. D. Leonhardt . 2016. “Urban Gardens Promote Bee Foraging Over Natural Habitats and Plantations.” Ecology and Evolution 6: 1304–1316. 26848387 10.1002/ece3.1941 PMC4730924

[ece372502-bib-0042] Kammerer, M. , J. F. Tooker , and C. M. Grozinger . 2020. “A Long‐Term Dataset on Wild Bee Abundance in Mid‐Atlantic United States.” Scientific Data 7: 240. 32686678 10.1038/s41597-020-00577-0 PMC7371858

[ece372502-bib-0044] Kearns, C. A. , D. W. Inouye , and N. M. Waser . 1998. “Endangered Mutualisms: The Conservation of Plant–Pollinator Interactions.” Annual Review of Ecology and Systematics 29: 83–112.

[ece372502-bib-0045] Kéry, M. , and J. A. Royle . 2016a. “Fitting Models Using the Bayesian Modeling Software BUGS and JAGS.” In Applied Hierarchical Modeling in Ecology. Vol. 1, 145–215. Elsevier.

[ece372502-bib-0046] Kéry, M. , and J. A. Royle . 2016b. “Hierarchical Models for Communities.” In Applied Hierarchical Modeling in Ecology. Vol. 1, 631–728. Elsevier.

[ece372502-bib-0048] Kéry, M. , and J. A. Royle . 2016c. “What Are Hierarchical Models and How Do We Analyze Them?” In Applied Hierarchical Modeling in Ecology. Vol. 1., 19–78. Elsevier.

[ece372502-bib-0050] Klein, A.‐M. , V. Boreux , F. Fornoff , A.‐C. Mupepele , and G. Pufal . 2018. “Relevance of Wild and Managed Bees for Human Well‐Being.” Current Opinion in Insect Science 26: 82–88. 29764666 10.1016/j.cois.2018.02.011

[ece372502-bib-0051] Kroll, A. J. , Y. Ren , J. E. Jones , et al. 2014. “Avian Community Composition Associated With Interactions Between Local and Landscape Habitat Attributes.” Forest Ecology and Management 326: 46–57.

[ece372502-bib-0053] Lawson, S. K. , P. Satyal , and W. N. Setzer . 2021. “The Volatile Phytochemistry of Seven Native American Aromatic Medicinal Plants.” Plants 10: 1061. 34070663 10.3390/plants10061061 PMC8229852

[ece372502-bib-0054] Lewthwaite, J. M. M. , T. M. Baiotto , B. V. Brown , et al. 2024. “Drivers of Arthropod Biodiversity in an Urban Ecosystem.” Scientific Reports 14: 390. 38172148 10.1038/s41598-023-50675-3 PMC10764344

[ece372502-bib-0056] Link, W. A. , and M. J. Eaton . 2012. “On Thinning of Chains in MCMC.” Methods in Ecology and Evolution 3: 112–115.

[ece372502-bib-0057] MacIvor, J. S. , and L. Packer . 2016. “The Bees Among Us: Modelling Occupancy of Solitary Bees.” PLoS One 11: e0164764. 27911954 10.1371/journal.pone.0164764 PMC5135037

[ece372502-bib-0059] MacKenzie, D. I. , J. D. Nichols , G. B. Lachman , S. Droege , J. A. Royle , and C. A. Langtimm . 2002. “Estimating Site Occupancy Rates When Detection Probabilities Are Less Than One.” Ecology 83: 2248–2255.

[ece372502-bib-0061] MacKenzie, D. I. , J. D. Nichols , J. A. Royle , K. H. Pollock , L. L. Bailey , and J. E. Hines . 2018. “Occupancy in Community‐Level Studies.” In Occupancy Estimation and Modeling, 2nd ed., 557–583. Elsevier.

[ece372502-bib-0062] Matteson, K. C. , J. S. Ascher , and G. A. Langellotto . 2008. “Bee Richness and Abundance in New York City Urban Gardens.” Annals of the Entomological Society of America 101: 140–150.

[ece372502-bib-0065] McKinney, A. M. , and K. Goodell . 2010. “Shading by Invasive Shrub Reduces Seed Production and Pollinator Services in a Native Herb.” Biological Invasions 12: 2751–2763.

[ece372502-bib-0066] Mola, J. M. , and N. M. Williams . 2025. “Bumble Bee Movement Ecology: Foraging and Dispersal Across Castes and Life Stages.” Annals of the Entomological Society of America 118, no. 3: 175–188. 40415971 10.1093/aesa/saaf010 PMC12095912

[ece372502-bib-0067] Mourguiart, B. , T. Couturier , Y. Braud , J. Mansons , D. Combrisson , and A. Besnard . 2021. “Multi‐Species Occupancy Models: An Effective and Flexible Framework for Studies of Insect Communities.” Ecological Entomology 46: 163–174.

[ece372502-bib-0068] Normandin, É. , N. J. Vereecken , C. M. Buddle , and V. Fournier . 2017. “Taxonomic and Functional Trait Diversity of Wild Bees in Different Urban Settings.” PeerJ 5: e3051. 28286711 10.7717/peerj.3051 PMC5344019

[ece372502-bib-0069] Ortiz, A. C. , S. C. Coster , and N. R. Ruppel . 2024. “Assessing Pollinator Richness on Urban‐Grown Mountain Mint (*Pycnanthemum* spp.) Populations.” Journal of Natural History 58: 2105–2120.

[ece372502-bib-0070] Osborne, J. L. , A. P. Martin , C. R. Shortall , et al. 2008. “Quantifying and Comparing Bumblebee Nest Densities in Gardens and Countryside Habitats.” Journal of Applied Ecology 45: 784–792.

[ece372502-bib-0071] Ostrom, R. B. J. , and K. L. Grayson . 2021. “First Record of *Hylaeus pictipes* Nylander, 1852 (Hymenoptera, Colletidae) in Virginia, United States of America.” Check List 17: 1375–1381.

[ece372502-bib-0072] Papanikolaou, A. D. , I. Kühn , M. Frenzel , and O. Schweiger . 2017. “Semi‐Natural Habitats Mitigate the Effects of Temperature Rise on Wild Bees.” Journal of Applied Ecology 54: 527–536.

[ece372502-bib-0199] Plummer, M. , 2003. “JAGS: A Program for Analysis of Bayesian Graphical Models Using Gibbs Sampling.” In Proceedings of the 3rd International Workshop on Distributed Statistical Computing, (Vol. 124, No. 125.10), 1–10.

[ece372502-bib-0301] R Core Team . 2023. R: A Language and Environment for Statistical Computing. R Foundation for Statistical Computing. https://www.R‐project.org/.

[ece372502-bib-0073] Rader, R. , B. G. Howlett , S. A. Cunningham , D. A. Westcott , and W. Edwards . 2012. “Spatial and Temporal Variation in Pollinator Effectiveness: Do Unmanaged Insects Provide Consistent Pollination Services to Mass Flowering Crops?” Journal of Applied Ecology 49: 126–134.

[ece372502-bib-0074] Rafferty, N. E. , P. J. CaraDonna , and J. L. Bronstein . 2015. “Phenological Shifts and the Fate of Mutualisms.” Oikos 124: 14–21. 25883391 10.1111/oik.01523 PMC4396844

[ece372502-bib-0075] Rasmussen, C. , M. S. Engel , and N. J. Vereecken . 2020. “A Primer of Host‐Plant Specialization in Bees.” Emerging Topics in Life Sciences 4, no. 1: 7–17. 32558903 10.1042/ETLS20190118

[ece372502-bib-0077] Richins, A. 2020. “Plant–Pollinator Associations in an Eastern Serpentine Savannah and the Effects of Overbrowsing.” Master's thesis, Virginia Commonwealth University.

[ece372502-bib-0079] Royle, J. A. , and R. M. Dorazio . 2009. “Conceptual and Philosophical Considerations in Ecology and Statistics.” In Hierarchical Modeling and Inference in Ecology, 1–26. Elsevier.

[ece372502-bib-0080] Royle, J. A. , and J. D. Nichols . 2003. “Estimating Abundance From Repeated Presence‐Absence Data or Point Counts.” Ecology 84: 777–790.

[ece372502-bib-0081] Ruppel, N. , S. Riley , E. Mumford , and B. Swedo . 2019. “Pollinator Visitation Frequency Associated With Native and Non‐Native Plants in a Mid‐Atlantic Piedmont (USA) Urban Garden.” Virginia Journal of Science 70: 15.

[ece372502-bib-0082] Schmack, J. M. , and M. Egerer . 2023. “Floral Richness and Seasonality Influences Bee and Non‐Bee Flower Interactions in Urban Community Gardens.” Urban Ecosystems 26: 1099–1112.

[ece372502-bib-0083] Schmitt, A. , R. Roy , and C. J. Carter . 2021. “Nectar Antimicrobial Compounds and Their Potential Effects on Pollinators.” Current Opinion in Insect Science 44: 55–63. 33771735 10.1016/j.cois.2021.03.004

[ece372502-bib-0084] Schwarz, G. 1978. “Estimating the Dimension of a Model.” Annals of Statistics 6: 461–464.

[ece372502-bib-0085] Setzer, W. N. , L. Duong , T. Pham , A. Poudel , C. Nguyen , and S. R. Mentreddy . 2021. “Essential Oils of Four Virginia Mountain Mint (*Pycnanthemum virginianum*) Varieties Grown in North Alabama.” Plants 10: 1397. 34371600 10.3390/plants10071397 PMC8309247

[ece372502-bib-0086] Silva, V. H. D. , I. N. Gomes , J. C. F. Cardoso , et al. 2023. “Diverse Urban Pollinators and Where to Find Them.” Biological Conservation 281: 110036.

[ece372502-bib-0087] Spiegelhalter, D. J. , N. G. Best , B. P. Carlin , and A. Van Der Linde . 2002. “Bayesian Measures of Model Complexity and Fit.” Journal of the Royal Statistical Society, Series B: Statistical Methodology 64: 583–639.

[ece372502-bib-0088] Sponsler, D. B. , and E. Z. Bratman . 2021. “Beekeeping in, of or for the City? A Socioecological Perspective on Urban Apiculture.” People and Nature 3: 550–559.

[ece372502-bib-0089] Theodorou, P. , K. Albig , R. Radzevičiūtė , et al. 2017. “The Structure of Flower Visitor Networks in Relation to Pollination Across an Agricultural to Urban Gradient.” Functional Ecology 31: 838–847.

[ece372502-bib-0090] Tobler, M. W. , A. Zúñiga Hartley , S. E. Carrillo‐Percastegui , and G. V. N. Powell . 2015. “Spatiotemporal Hierarchical Modelling of Species Richness and Occupancy Using Camera Trap Data.” Journal of Applied Ecology 52: 413–421.

[ece372502-bib-0091] Udy, K. L. , H. Reininghaus , C. Scherber , and T. Tscharntke . 2020. “Plant–Pollinator Interactions Along an Urbanization Gradient From Cities and Villages to Farmland Landscapes.” Ecosphere 11: e03020.

[ece372502-bib-0092] United Nations Department of Economic and Social Affairs . 2023. The Sustainable Development Goals Report 2023: Special Edition. United Nations. 10.18356/9789210024914.

[ece372502-bib-0093] US Census Bureau . 2022. https://www.census.gov.

[ece372502-bib-0094] Vanderplanck, M. , N. J. Vereecken , L. Grumiau , et al. 2017. “The Importance of Pollen Chemistry in Evolutionary Host Shifts of Bees.” Scientific Reports 7: 43058. 28216663 10.1038/srep43058 PMC5316986

[ece372502-bib-0095] Watanabe, S. 2013. “A Widely Applicable Bayesian Information Criterion.” Journal of Machine Learning Research 14: 867–897.

[ece372502-bib-0096] Weakley, A. S. , B. Crowder , J. F. Townsend , and J. C. Ludwig . 2012. Flora of Virginia. 1st ed. Botanical Research Institute of Texas Press.

[ece372502-bib-0097] Whittaker, R. H. 1960. “Vegetation of the Siskiyou Mountains, Oregon and California.” Ecological Monographs 30: 279–338.

[ece372502-bib-0098] Williams, N. M. , and R. Winfree . 2013. “Local Habitat Characteristics but Not Landscape Urbanization Drive Pollinator Visitation and Native Plant Pollination in Forest Remnants.” Biological Conservation 160: 10–18.

[ece372502-bib-0099] Zaninotto, V. , A. Perrard , O. Babiar , A. Hansart , C. Hignard , and I. Dajoz . 2021. “Seasonal Variations of Pollinator Assemblages Among Urban and Rural Habitats: A Comparative Approach Using a Standardized Plant Community.” Insects 12: 199. 33673434 10.3390/insects12030199 PMC7996759

