# Supplementary material for: Influence of Environmental Covariates on Pollinator Community Occupancy, Detection, and Richness Across Urban Gardens in Richmond, Virginia, USA
Source: Ecol Evol. 2025 Nov 17;15(11):e72502. doi: 10.1002/ece3.72502 (PMC12623005; doi:10.1002/ece3.72502)
Supplement: Supplementary file 1 — Appendices S1–S6: ece372502‐sup‐0001‐AppendicesS1‐S6.zip. [file ECE3-15-e72502-s001.zip › Appendix_tables.docx]

| *Appendix Tables*  Appendix Table A1.1. Summary of visual pollinator surveys conducted at 50 gardens around the Greater Richmond, VA area, by site (Garden Name), internal numerical identifier (Code), the number of unique species detected at the garden (# of Species), Total Detections (including replicate detections of the same species), and whether surveys were conducted on *Pycnanthemum muticum*, *Liatris spicata*, or both (Plant). | | | | |
| --- | --- | --- | --- | --- |
| Garden Name | Code | # of Species | Total Detections | Plant |
| Private | 1 | 4 | 9 | Both |
| Private | 2 | 1 | 3 | *P. muticum* |
| Private | 3 | 3 | 8 | Both |
| Private | 4 | 0 | 0 | *P. muticum* |
| Armour House Park | 5 | 5 | 7 | Both |
| Private | 6 | 3 | 3 | *L. spicata* |
| Private | 7 | 4 | 10 | *P. muticum* |
| Private | 8 | 1 | 1 | *P. muticum* |
| Private | 9 | 4 | 7 | Both |
| Bryan Park | 10 | 5 | 11 | Both |
| Chimborazo Park | 11 | 3 | 7 | *P. muticum* |
| Covenant Woods | 12 | 4 | 14 | Both |
| Private | 13 | 4 | 10 | *P. muticum* |
| Private | 14 | 2 | 6 | *P. muticum* |
| Private | 15 | 3 | 6 | *L. spicata* |
| Private | 16 | 4 | 10 | *P. muticum* |
| Private | 17 | 2 | 7 | Both |
| Private | 18 | 3 | 11 | *L. spicata* |
| Private | 19 | 1 | 5 | *P. muticum* |
| Private | 20 | 2 | 9 | Both |
| Hanover Arts & Activities Center | 21 | 1 | 3 | Both |
| Private | 22 | 3 | 9 | *P. muticum* |
| Private | 23 | 2 | 4 | *P. muticum* |
| Private | 24 | 1 | 3 | *L. spicata* |
| Private | 25 | 3 | 7 | *P. muticum* |
| Private | 26 | 2 | 6 | *P. muticum* |
| Private | 27 | 1 | 5 | *P. muticum* |
| Private | 29 | 3 | 7 | Both |
| Private | 30 | 2 | 3 | *L. spicata* |
| Private | 31 | 4 | 10 | *P. muticum* |
| Private | 32 | 3 | 4 | Both |
| Lewis Ginter Botanical Garden | 33 | 4 | 6 | Both |
| Linwood Holton Elementary | 34 | 1 | 1 | *L. spicata* |
| Private | 35 | 3 | 10 | *P. muticum* |
| Private | 36 | 4 | 10 | *P. muticum* |
| Private | 37 | 3 | 12 | Both |
| Private | 38 | 3 | 5 | Both |
| Private | 39 | 4 | 8 | *L. spicata* |
| Private | 40 | 5 | 14 | Both |
| Private | 41 | 4 | 7 | *P. muticum* |
| Randolph-Macon College Garden | 42 | 4 | 7 | Both |
| Private | 43 | 2 | 8 | Both |
| Private | 44 | 3 | 9 | Both |
| Private | 45 | 4 | 12 | *P. muticum* |
| Short Pump Park | 46 | 4 | 8 | Both |
| Private | 47 | 2 | 2 | *L. spicata* |
| Unity at Bon Air | 49 | 5 | 15 | *P. muticum* |
| Wawa Ashland | 50 | 1 | 4 | *L. spicata* |
| Woodlake Development | 51 | 2 | 6 | *L. spicata* |
| Private | 52 | 3 | 12 | Both |

| Appendix Table A1.2. Summary of visual insect surveys conducted at 50 gardens around the Greater Richmond, VA area, by lowest identifiable taxonomic level (Taxon), shorthand used in code and figures (Abbreviation), the number of distinct gardens at which the taxon was detected (# of Sites), Total Detections (including replicate detections in the same garden), and whether the taxon was detected on *Pycnanthemum muticum*, *Liatris spicata*, or both (Plant). | | | | |
| --- | --- | --- | --- | --- |
| Taxon | Abbreviation | # of Sites | Total Detections | Plant |
| *Bombus* spp. | Bombus spp. | 28 | 81 | Both |
| *Xylocopa virginica* | XyVi | 18 | 28 | Both |
| *Apis mellifera* | Apis | 35 | 123 | Both |
| *Halictidae* family | Halictidae | 33 | 84 | Both |
| *Coelioxys octodentatus* | CoOc | 6 | 7 | Both |
| *Anthidium oblongatum* | AnOb | 1 | 2 | Both |
| All wasps (Vespoidea) | Wasps | 9 | 15 | *P. muticum* |
| *Erynnis horatius* | ErHo | 1 | 1 | *P. muticum* |
| *Macrosiagon limbatum* | MaLi | 3 | 5 | *P. muticum* |
| *Halyomorpha halys* | HaHa | 1 | 2 | *P. muticum* |
| *Pieris rapae* | PiRa | 1 | 1 | *L. spicata* |
| *Atalopedes campestris* | AtCa | 4 | 7 | *L. spicata* |
| *Megachile* spp. | Megachile spp. | 3 | 4 | *L. spicata* |
| *Epilachna borealis* | EpBo | 1 | 1 | *L. spicata* |

| Appendix Table A4. Off-diagonal matrix of pairwise correlations (Pearson’s *r*) between survey covariates tested as potential predictors in the detection sub-model of multi-species occupancy models for the pollinator community around Richmond, VA. Covariates with $\left\vert r \right\vert>0.6$ were considered highly correlated and were not combined in the same model. | | | | | |
| --- | --- | --- | --- | --- | --- |
|  | Time of Day | Julian Date | Temperature | Light Level | Bloom Richness |
| Time of Day | - |  |  |  |  |
| Julian Date | -0.12 | - |  |  |  |
| Temperature | 0.49 | -0.59 | - |  |  |
| Light Level | 0.12 | -0.31 | 0.35 | - |  |
| Bloom Richness | -0.35 | 0.24 | -0.17 | -0.01 | - |

| Appendix Table A5. Summary of variable selection process. All models shown here had constant occupancy for a given taxon, but occupancy could vary across taxa. Shown are the stage of the forward step-wise selection process (Step), symbolic notation for a particular model following R formula notation (Detection sub-model), the number of coefficients in the model for which the 95% CrI did not cross 0 (# Influential coefficients), the number of additional coefficients (Influential new terms), compared to the reference model from the previous selection step, for which the 95% CrI did not cross 0, and additional comments about the selection decisions made (Notes). | | | | |
| --- | --- | --- | --- | --- |
| Step | Detection sub-model | # Influential coefficients | Influential new terms | Notes |
| Null | p ~ plant | 4 | 4 |  |
| Round 1 | p ~ plant + time | 6 | 2 |  |
| 1 main effect | p ~ plant + jday | 5 | 2 |  |
|  | p ~ plant + temp | 8 | 3 |  |
|  | p ~ plant + lux | 8 | 4 |  |
|  | p ~ plant + bloomr | 5 | 0 | Dropped |
|  | p ~ plant + ud | 6 | 2 |  |
|  | p ~ plant + ga | 5 | 1 | Dropped |
| 1 interaction | p ~ plant * time | 17 | 2 | New base |
|  | P ~ plant * jday | 5 | 0 | Dropped |
|  | p ~ plant * temp | 10 | 2 |  |
|  | p ~ plant * lux | 7 | 0 | Dropped |
|  | p ~ plant * ud | 8 | 1 | Dropped |
| quadratics | p ~ plant + time^2 | 7 | 1 | Dropped |
|  | p ~ plant + jday^2 | 5 | 0 | Dropped |
|  | p ~ plant + temp^2 | 8 | 2 | Dropped in r3 |
|  | p ~ plant + bloomr^2 | 5 | 0 | Dropped |
|  | p ~ plant + ud^2 | 6 | 0 | Dropped |
|  | p ~ plant + ga^2 | 5 | 0 | Dropped |
|  | p ~ (plant * time) + (time * jday) | 9 | 0 | Dropped |
| Round 2 | p ~ plant * time + jday | 20 | 2 |  |
| 2 variables | P ~ plant * time + temp | 12 | 2 |  |
|  | p ~ plant * time + lux | 18 | 3 |  |
|  | p ~ plant * time + ud | 21 | 2 | New base |
| 2 var interaction | p ~ (plant * time) + (time * lux) | 17 | 0 | Dropped |
|  | p ~ (plant * time) + (time * ud) | 22 | 0 | Dropped |
|  | p ~ (plant * time) + (time * jday) | 9 | 0 | Dropped |
| Round 3 | p ~ (plant * time) + ud + jday | 22 | 3 |  |
| 3 variables | p ~ (plant * time) + ud + temp | 14 | 1 | Dropped |
|  | p ~ (plant * time) + ud + lux | 20 | 3 |  |
| 3 var interaction | p ~ (plant * time) + (ud * jday) | 22 | 2 | New base |
| Round 4 | p ~ (plant * time) + ud + jday + lux | 21 | 3 |  |
|  | p ~ (plant * time) + (ud * jday) + lux | 24 | 3 | Selected |
|  | p ~ (plant * time) + (ud * lux) + jday | 21 | 3 |  |
|  | p ~ (plant * time) + (jday * lux) + ud | 23 | 4 |  |
| p = detection probability; plant = plant species; time = survey start time; jday = Julian date; temp = temperature; lux = light level; bloomr = bloom richness; ud = distance to urban center; ga = garden area; + combination of main effects only; * = interaction | | | | |

| Appendix Table A6. Posterior probabilities of being positive for species-specific detection coefficients from final model of multi-species occupancy analysis of pollinators around Richmond, VA. Posterior probabilities are the proportion of posterior samples, across all MCMC chains that were > 0. Larger values indicate a greater probability of a positive effect, values closer to 0 indicate a greater probability of a negative effect, and values around 0.5 indicate little evidence of a meaningful (non-zero) effect. All model terms (columns) pertain to the detection sub-modal. For each detected taxon (rows), columns shown are the change (compared to *P. muticum*) in detection for surveys conducted on *L. spicata*, the main effect of time of day (Start time) , the main effect of distance from urban center (Urban distance), the main effect of Julian date, the main effect of light level (Light level (lux)), the interaction between detection on *L. spicata* and time of day (Flower x time), and the interaction between distance from urban center and Julian date (Urban dist. x Julian date). | | | | | | | |
| --- | --- | --- | --- | --- | --- | --- | --- |
| Taxon | *L. spicata* | Start time | Urban distance | Julian date | Light level (lux) | Flower x time | Urban dist. x Julian date |
| Bombus spp. | 0.15 | 1.00 | 1.00 | 0.01 | 0.96 | 0.77 | 0.33 |
| XyVi | 0.39 | 0.98 | 0.62 | 0.37 | 0.99 | 0.01 | 0.18 |
| Apis | 0.00 | 1.00 | 0.41 | 0.02 | 0.35 | 0.02 | 0.02 |
| Halictidae | 0.94 | 1.00 | 0.00 | 0.83 | 1.00 | 0.00 | 0.00 |
| CoOc | 0.05 | 0.98 | 0.07 | 0.30 | 0.98 | 0.03 | 0.08 |
| AnOb | 0.25 | 0.94 | 0.31 | 0.24 | 0.89 | 0.13 | 0.22 |
| Vespoidea | 0.01 | 0.97 | 0.78 | 0.53 | 0.86 | 0.15 | 0.15 |
| ErHo | 0.14 | 0.96 | 0.51 | 0.20 | 0.75 | 0.17 | 0.19 |
| MaLi | 0.03 | 0.99 | 0.24 | 0.24 | 0.93 | 0.15 | 0.24 |
| HaHa | 0.21 | 0.95 | 0.51 | 0.21 | 0.84 | 0.17 | 0.19 |
| PiRa | 0.37 | 0.97 | 0.57 | 0.16 | 0.91 | 0.20 | 0.11 |
| AtCa | 0.98 | 0.98 | 0.61 | 0.26 | 0.79 | 0.34 | 0.17 |
| Megachile spp. | 0.81 | 0.96 | 0.22 | 0.21 | 0.92 | 0.03 | 0.33 |
| EpBo | 0.41 | 0.95 | 0.49 | 0.17 | 0.83 | 0.05 | 0.16 |
| Unknown1 | 0.30 | 0.96 | 0.44 | 0.28 | 0.84 | 0.18 | 0.20 |

*Appendix Figure Captions*

Appendix Figure A3.1. Joint posterior distribution of the observed and expected deviances for final multi-species occupancy model, *ψ* ~ 1, *p* ~ (plant_species * time) + (urban distance * Julian date) + light level, for the pollinator community around Richmond, VA, U.S.A.. Perfect agreement between the observed data and the posterior predictive distribution would result in points falling exactly along the 1-to-1 line (black reference line). Points below the reference line (dark gray) constitute a graphical representation of the Bayesian *p*-value (0.61).

Appendix Figure A3.2. Diagnostic plot for final model displaying posterior mean deviance contributions (*d*^2^) by all sites (A), sites where only *P. muticum* were surveyed (B), sites where only *L. spicata* were surveyed (C) and sites where surveys were conducted on both plant species (D). Sites on the x-axes have been ordered by distance to urban center (shortest on the left to farthest on the right) to examine any additional patterns related to that covariate. Box plots summarize the deviance contributions of 14 detected taxa and 1 undetected taxon at each site, with black bands indicating the median, gray boxes covering the first to third quartile, whiskers extending to 1.5 times the interquartile range, and circles indicating outliers beyond that range. Values of *d*^2^ further away from zero indicate less agreement between the model and observed data.

Appendix Figure A3.3. Diagnostic plot for final model displaying posterior mean deviance contributions (*d*^2^) by 14 detected taxa and 1 undetected taxon across all sites (A, n = 50 sites), sites where only *P. muticum* were surveyed (B, n = 20 sites), sites where only *L. spicata* were surveyed (C, n = 10 sites) and sites where surveys were conducted on both plant species (D, n = 20 sites). Taxa on the x-axes have all been sorted identically, with the most detected taxa to the left and the least detected to the right. Box plots summarize the deviance contributions, with black bands indicating the median, gray boxes covering the first to third quartile, whiskers extending to 1.5 times the interquartile range, and circles indicating outliers beyond that range. Values of *d*^2^ further away from zero indicate less agreement between the model and observed data.
