# Supplementary material for: Influence of Environmental Covariates on Pollinator Community Occupancy, Detection, and Richness Across Urban Gardens in Richmond, Virginia, USA
Source: Ecol Evol. 2025 Nov 17;15(11):e72502. doi: 10.1002/ece3.72502 (PMC12623005; doi:10.1002/ece3.72502)
Supplement: Supplementary file 1 — Appendices S1–S6: ece372502‐sup‐0001‐AppendicesS1‐S6.zip. [file ECE3-15-e72502-s001.zip › ece372502-sup-0001-AppendicesS1-S6.docx]

**Appendices**

*Appendix S1: Raw data summaries*

Table A1.1

Table A1.2

*Appendix S2: Review of Bayesian Variable Selection and Description of Approach*

Model selection in Bayesian analyses remains an active area of research in statistical literature (Hooten and Hobbs 2015) and there is currently no consensus approach analogous to AIC and its extensions that are widely applied to maximum likelihood methods (Burnham and Anderson 2002). This is especially true of specific, relatively recent, applications of Bayesian methods like MSOMs (Broms et al. 2016). Inasmuch as the choice of model selection procedure may influence the results of an analysis and inferences based on those results, and in the interest of transparency in our decision-making process, we briefly review possible approaches to Bayesian variable selection, their applicability to our research objectives, and then provide a detailed description of the approach taken in this analysis.

*Review of Bayesian Variable Selection*

Regarding information criteria, AIC and the similar Bayesian Information Criterion (BIC; Schwarz 1978) are based on maximum likelihood theory, and while some authors have implemented versions of them in a Bayesian context (Link and Barker 2010, Kirsch and Peterson 2014), they are generally not considered appropriate for Bayesian hierarchical models (Hooten and Hobbs 2015, Broms et al. 2016). Deviance Information Criterion (DIC) (Spiegelhalter et al. 2002) has been found to perform well for some types of Bayesian models, but has been criticized when used for hierarchical or random effects models with latent variable structure (Hooten and Hobbs 2015, Broms et al. 2016). More recently, WAIC (variously rendered as Watanabe-Akaike Information Criterion or Widely Applicable Information Criterion; Watanabe 2013) has gained attention in ecological literature (e.g. Hooten and Hobbs 2015), but to our knowledge, its performance has yet to be systematically evaluated for MSOMs (however, for examples of WAIC in MSOMs applied in specific contexts, see Broms et al. 2016, McClenaghan et al. 2020, Doser et al. 2022). Model averaging is another approach to multimodel inference that can be implemented using various information criteria (Burnham and Anderson 2002) but in a Bayesian context is more often implemented via Bayes factors (Kass and Raftery 1995) or indicator variables for the inclusion of particular covariates (Kuo and Mallick 1998). However, model averaging focuses on predictions of response variables while incorporating model selection uncertainty; the effects of specific covariates may often be obscured because the models being averaged include different covariates. The model weights from Bayes factors or indicator variables can themselves be used as a basis for model comparison, but thresholds for distinguishing between models with similar weights are less clearcut than for information criteria (Link and Barker 2010). K-fold cross-validation is generally considered to perform well for a wide range of Bayesian models (Gelman et al. 2014, Hooten and Hobbs 2015, Broms et al. 2016), but it can be computationally intensive because it requires repeatedly fitting models to different subsets of data and, because it involves withholding some data for model validation, may be inefficient or even implausible for ecological data sets that are often sparse. Additionally, cross-validation methods were developed for the purpose of out-of-sample prediction, not parameter estimation or within-sample prediction per se. Some authors have argued against the practice of variable selection in a Bayesian context entirely (Gelman et al. 2014).

*Implemented Approach*

With no clear model selection procedure that is both well-supported in the literature for application to MSOMs and well-suited to our objectives, we instead employed a model selection approach that was more heuristic than procedural. We considered a covariate influential for a given species if the central 95% credible interval (CrI) for the associated species-specific regression coefficient did not cross 0, and used a forward selection process to test each covariate individually and retained covariates that were influential for multiple species. Royle et al. (2014) noted that such an approach was analogous to traditional hypothesis testing, albeit in the context of spatial capture-recapture models rather than MSOMs. A Bayesian interpretation, without reference to significance testing, would be that if the CrI does not cross 0 there is a very high probability (> 97.5%) that the covariate has a meaningful (non-zero) effect, with the posterior mean—or other measure of central tendency—as a summary of the magnitude of that effect. Broms et al. (2016) included a similar model focusing only on covariates affecting multiple species when they used data on the fish community of the South Platte River basin in Colorado to compare several model selection approaches, specifically in the context of MSOMs, and found that the model performed well across several model selection procedures.

Starting with the occupancy sub-model (MacKenzie et al. 2018*b*), we added each covariate individually, and examined the posterior distributions of associated species-specific regression coefficients, while keeping the detection sub-model consistent with only the plant type main effect (p ~ plant). After selecting covariates for occupancy, this was held consistent while testing covariates on the detection sub-model. If the covariate was influential for two or more species, we deemed the covariate to be influential on (at least a portion of) the larger community, thus we retained the covariate in subsequent steps. If one or no species had a regression coefficient for which the CrI did not cross 0, the covariate was excluded from subsequent steps. In the initial step with only a single covariate in addition to the plant term on detection probability, we also tested quadratic versions of covariates to examine potentially plateauing of effects (especially for distance or areal metrics). Whenever a quadratic term was added to the model, the linear version of that term was also included. As above, if the CrI for the quadratic term did not cross 0 for 2 or more species, it was retained for future steps, otherwise it was excluded. Even if a quadratic term were excluded, the linear term for that covariate might still be retained. However, if the linear term were excluded, then the quadratic term was as well. Next, we selected the covariate that was influential for the most species and used that as our next base model. From the remaining covariates that were not excluded in the previous step, we added them one at a time to this new base model to find the covariate that was influential on the next most species. When combining two or more covariates, we first added the new covariate as a main effect only, but then took an additional step of adding an interaction. Similar to quadratics, any model with an interaction also included the main effects of the potentially interacting terms. The same rules were used to retain or exclude the interaction term, and a main effect might be retained even if an interaction were excluded, but if a main effect were excluded then an interaction with that covariate would not be retained. When multiple terms were combined in the same model, sometimes there was a change in the number of taxa for which a previously retained term was influential. For example, when the detection model included only main effects for plant type and survey start time (p ~ plant + time), there were a total of 6 influential species-specific coefficients, 4 taxa related to plant type and two related to time. When an additional interaction term was included (p ~ plant * time or, equivalently, p ~ plant + time + plant:time), there were a total of 17 influential coefficients: the same 4 for the main effect of plant type, 11 for the main effect of start time, and 2 for the interaction between plant and time. In such cases, we selected the next base model as the one that yielded the most influential coefficients across all terms, not just the most recently added term. This process was repeated until all variables (and related terms like quadratics or interactions) had either been included or eliminated.

*Appendix S3: Goodness-of-Fit and Model Assessment*

*Review of Model Fit Assessment and Multi-species Occupancy Modeling*

Assessing fit of Bayesian models usually employs the posterior predictive distribution and some type of Bayesian *p*-value (Gelman et al. 1996, Broms et al. 2016, Kéry and Royle 2016*b*). At each MCMC iteration, new data is simulated from the current values of each parameter and some statistic is calculated on both the simulated and observed data. Comparing these statistics across all MCMC samples yields a Bayesian *p*-value. Similar to variable selection, however, there is little consensus in the literature on what specific statistic to employ, particularly for MSOMs (Broms et al. 2016). There is general agreement that data with a binary response needs to be aggregated prior to goodness-of-fit testing (GoF) (McCullagh and Nelder 1998, Kéry and Royle 2016*d*). For single-species occupancy models, MacKenzie and Bailey (2004) proposed a GoF procedure based on a χ^2^ statistic (i.e. based on Pearson’s residuals) after aggregating response data, and some authors have adapted this approach to MSOMs (Carrillo-Rubio et al. 2014, Kroll et al. 2014, Tobler et al. 2015). However, Broms et al. (2016) note that hierarchical occupancy models are not expected to follow a χ^2^ distribution even after aggregation. The Freeman-Tukey statistic has been suggested as an alternative for Bayesian *p*-values (Kéry and Royle 2016*c*, Doser et al. 2022) but it is also based on a χ^2^ distribution (Read 1993) and its applicability to multi-level MSOMs is similarly unclear. Here, we instead follow Broms et al. (2016) in basing our Bayesian *p*-values on deviance, due to the strong theoretical link between deviance and Kullback-Leibler divergence, and the wide acceptance of deviance as a measure of model fit across a range of model types (Spiegelhalter et al. 2002). Perfect fit would be indicated by a Bayesian *p*-value = 0.5, and we considered *P*-values > 0.9 or < 0.1 to indicate unacceptable fit. We also assessed fit visually by plotting the posterior predictive deviance against the observed deviance (Brooks et al. 2000, Kéry and Royle 2016*b*), and by plotting deviance contributions by species, sites, and site covariates (Broms et al. 2016, McClenaghan et al. 2020). However, because our formulation of the likelihood (below) aggregated observations over site replicates (i.e. the number of detections of each species at each site), we were unable to plot deviance contributions vs. survey covariates. We present Bayesian *p*-value and diagnostic plots only for our final model.

*Integrated Likelihood*

Adapted from Broms et al. (2016) to incorporate data augmentation.

*Notation*

*i*: indexes species, up to *M* possible species

*j*: indexes sites, up to *J* total sites

*k*: indexes replicate surveys within a site, up to *K_j_* replicate surveys

*s*: indexes MCMC samples from the posterior distribution, up to S posterior samples

*Ω* = probability of community membership

*w_i_* = binary indicator that takes a value of 1 if species *i* is in the community, otherwise 0

*ψ_ij_* = probability that species *i* is present at site *j*, given that it is in the community

*z_ij_* = binary indicator that takes a value of 1 if species *i* is present at site *j*, otherwise 0

*p_ijk_* = probability that species *i* is detected at site *j* on replicate survey *k*, given that it is in the community and present at site *j*

*y_ijk_* = binary indicator that takes a value of 1 if species *i* was detected at site *j* on replicate survey *k*, otherwise 0

$I_{\{\sum_{k=1}^{K_{j}} y_{ijk}>0\}}$: is an indicator function that takes a value of 1 if species *i* was detected at least once at site *j*. Otherwise it takes a value of 0.

[***y****_ij_* | *ψ*_ij_, ***p****_ij_*] : is the integrated likelihood, when the binary detections of species *i* at site *j* on replicate survey *k* are aggregated to counts of detection of species *i* at site *j* (across all *K_j_* replicates).

*D* = model deviance

*d^2^* = contribution of an individual set of observations to the overall model deviance

*d* = deviance residual

| $\left[ y_{ij} \right\vert\Omega,\psi_{ij},p_{ij}]= I_{\left\{ \sum_{k=1}^{K_{j}} y_{ijk}>0 \right\}}\left( \Omega\psi_{ij}\prod_{k=1}^{K_{j}} p_{ijk}^{y_{ijk}}\left( 1-p_{ijk} \right)^{\left( 1-y_{ijk} \right)} \right)+$ $\left( 1-I_{\left\{ \sum_{k=1}^{K_{j}} y_{ijk}>0 \right\}} \right)\left( \Omega\left( \psi_{ij}\prod_{k=1}^{K_{j}} \left( 1-p_{ijk} \right) \right)+\Omega\left( 1-\psi_{ij} \right)+\left( 1-\Omega\right) \right)$ | Eq. A3.1 |
| --- | --- |

If species *i* was detected at least once at site *j* (meaning it must be in the community and present at the site), the second line drops out and only the first line contributes to the likelihood. If species *i* was never detected at site *j*, the first line drops out and the second line comprises the probabilities that A) species *i* is in the community and present at site *j* but went completely undetected; or B) species *i* is in the community but not present at site *j*; or C) species *i* is not in the community at all.

*Deviance, Bayesian p-value, and Diagnostic Plots*

For each MCMC sample, the observed deviance ($D^{(s)}$) and the posterior predictive deviance ($\tilde{D}^{(s)}$) were calculated from the parameter values for that sample. Note that, here, ^(^*^s^*^)^ does not denote exponentiation, but indexes the *s* = 1,…,*S* iterations of MCMC sampling, with superscript used to distinguish from the *i*, *j* (subscript) indexing of the likelihood. The individual contribution to the deviance of each set of observations of species *i* at site *j* can then be specified as

| ${d^{2}}^{(s)}=-2log\left[ y_{ij} \right\vert\Omega^{(s)},\psi_{ij}^{(s)},p_{ij}^{(s)}]$ | Eq. A3.2 |
| --- | --- |

and summing over all sites and all species gives the total observed deviance (for that iteration):

| $D^{(s)}=-2\sum_{i=1}^{M} \sum_{j=1}^{J} log\left[ y_{ij} \right\vert\Omega^{(s)},\psi_{ij}^{(s)},p_{ij}^{(s)}]=\sum_{i=1}^{M} \sum_{j=1}^{J} {d^{2}}^{(s)}$ | Eq. A3.3 |
| --- | --- |

The posterior predictive distribution was generated by simulating new data at each iteration as

$$\tilde{w}_{i}^{\left( s \right)}\sim Bern\left( \Omega^{\left( s \right)} \right)$$

$$\tilde{z}_{ij}^{\left( s \right)}\sim Bern\left( \tilde{w}_{i}^{\left( s \right)}\psi_{ij}^{\left( s \right)} \right)$$

$$\tilde{y}_{ijk}^{\left( s \right)}\sim Bern\left( \tilde{z}_{ij}^{\left( s \right)}p_{ijk}^{\left( s \right)} \right)$$

and the simulated observations were then used to calculate the posterior predictive deviance using Eqs. A3.2 and A3.3.

$${\tilde{d}^{2}}^{\left( s \right)}=-2log\left[ {\tilde{\boldsymbol{y}}}_{ij}^{\left( s \right)} \right|\Omega^{\left( s \right)},\psi_{ij}^{\left( s \right)},p_{ij}^{\left( s \right)}]$$

$$\tilde{D}^{(s)}=\sum_{i=1}^{M} \sum_{j=1}^{J} {\tilde{d}^{2}}^{(s)}$$

We then calculated the Bayesian *p*-value as the proportion of times, over all MCMC iterations, that $D^{(s)}> \tilde{D}^{(s)}$. Calculations were adapted from Broms et al. (2016), and they, as well as McClenaghan et al. (2020), created diagnostic plots using the posterior means of what we have called $d^{2}$ and we follow that approach as well. However, we note that, although the previous authors referred to these as “deviance residuals,” strictly they are the square of the deviance residuals (hence our notation $d^{2}$) (Spiegelhalter et al. 2002). Both can be used to identify areas where the model does not fit (with values closer to 0 indicating closer agreement between the model and the data), but the (signed) deviance residuals can also identify the direction of lack-of-fit (e.g. overpredicting or underpredicting), whereas the squared residuals cannot because they are always positive.

Figure A3.1

Figure A3.2

Figure A3.3

*Appendix S4: Correlation Tests Between Covariates*

Table A4

Our analysis included only 2 site covariates, distance from urban center and garden area, which had a correlation of *r* = 0.54. Because site covariates and survey covariates had different dimensions (50 x 1 vectors for site covariates, 50 x 10 matrices for survey covariates), it was not possible to calculate *r* comparing site to survey covariates.

*Appendix S5: Variable selection*

Table A5

*Appendix S6: Posterior probabilities of coefficient estimates*

Table A6
