# Supplementary material for: Influence of Environmental Covariates on Pollinator Community Occupancy, Detection, and Richness Across Urban Gardens in Richmond, Virginia, USA
Source: Ecol Evol. 2025 Nov 17;15(11):e72502. doi: 10.1002/ece3.72502 (PMC12623005; doi:10.1002/ece3.72502)
Supplement: Supplementary file 1 — Appendices S1–S6: ece372502‐sup‐0001‐AppendicesS1‐S6.zip. [file ECE3-15-e72502-s001.zip › Appendix Literature Cited.docx]

**Appendix Literature Cited**

Brooks, S. P., E. A. Catchpole, and B. J. T. Morgan. 2000. Bayesian animal survival estimation. Statistical Science 15:357–376.

Burnham, K. P., and D. R. Anderson. 2002. Model selection and multimodel inference: a practical information-theoretic approach. 2nd edition. Springer, New York, NY, USA.

Gelman, A., J. B. Carlin, H. S. Stern, D. B. Dunson, A. Vehtari, and D. B. Rubin. 2014. Bayesian data analysis. Third edition. Chapman & Hall/CRC texts in statistical science, CRC Press, Boca Raton.

Gelman, A., X.-L. Meng, and H. Stern. 1996. Posterior predictive assessment of model fitness via realized discrepancies. Statistica Sinica 6:733–760.

Kass, R. E., and A. E. Raftery. 1995. Bayes factors. Journal of the American Statistical Association 90:773–795.

Kéry, M., and J. A. Royle. 2016*c*. What are hierarchical models and how do we analyze them? Pages 19–78 *in*. Applied Hierarchical Modeling in Ecology. Volume 1. Elsevier, London, UK.

Kirsch, J. E., and J. T. Peterson. 2014. A multi‐scaled approach to evaluating the fish assemblage Structure within southern Appalachian streams. Transactions of the American Fisheries Society 143:1358–1371.

Kuo, L., and B. Mallick. 1998. Variable selection for regression models. Sankhyā: The Indian Journal of Statistics, Series B (1960-2002) 60:65–81.

Link, W. A., and R. J. Barker. 2010. Multimodel inference. Pages 127-159 *in*. Bayesian inference: with ecological applications. First edition. Elsevier Science & Technology, San Diego, CA, USA.

MacKenzie, D. I., and L. L. Bailey. 2004. Assessing the fit of site-occupancy models. Journal of Agricultural, Biological, and Environmental Statistics 9:300–318.

MacKenzie, D. I., J. D. Nichols, J. A. Royle, K. H. Pollock, L. L. Bailey, and J. E. Hines. 2018*a*. Occupancy in community-level studies. Pages 557–583 *in*. Occupancy Estimation and Modeling. 2^nd^ edition. Elsevier, San Diego, CA, USA.

McClenaghan, B., Z. G. Compson, and M. Hajibabaei. 2020. Validating metabarcoding-based biodiversity assessments with multi-species occupancy models: A case study using coastal marine eDNA. PLOS ONE 15:e0224119.

McCullagh, P., and J. A. Nelder. 1998. Generalized linear models. 2nd edition. Monographs on statistics and applied probability 37, Chapman & Hall/CRC, Boca Raton, FL, USA.

Read, C. B. 1993. Freeman—Tukey chi-squared goodness-of-fit statistics. Statistics & Probability Letters 18:271–278.

Royle, J. A., R. B. Chandler, R. Sollmann, and B. Gardner. 2014. Model selection and assessment. Pages 219–243 *in*. Spatial Capture-recapture. Elsevier, Boston, MA, USA.
