# Supplementary figures and images for: Influence of Environmental Covariates on Pollinator Community Occupancy, Detection, and Richness Across Urban Gardens in Richmond, Virginia, USA
Source: Ecol Evol. 2025 Nov 17;15(11):e72502. doi: 10.1002/ece3.72502 (PMC12623005; doi:10.1002/ece3.72502)

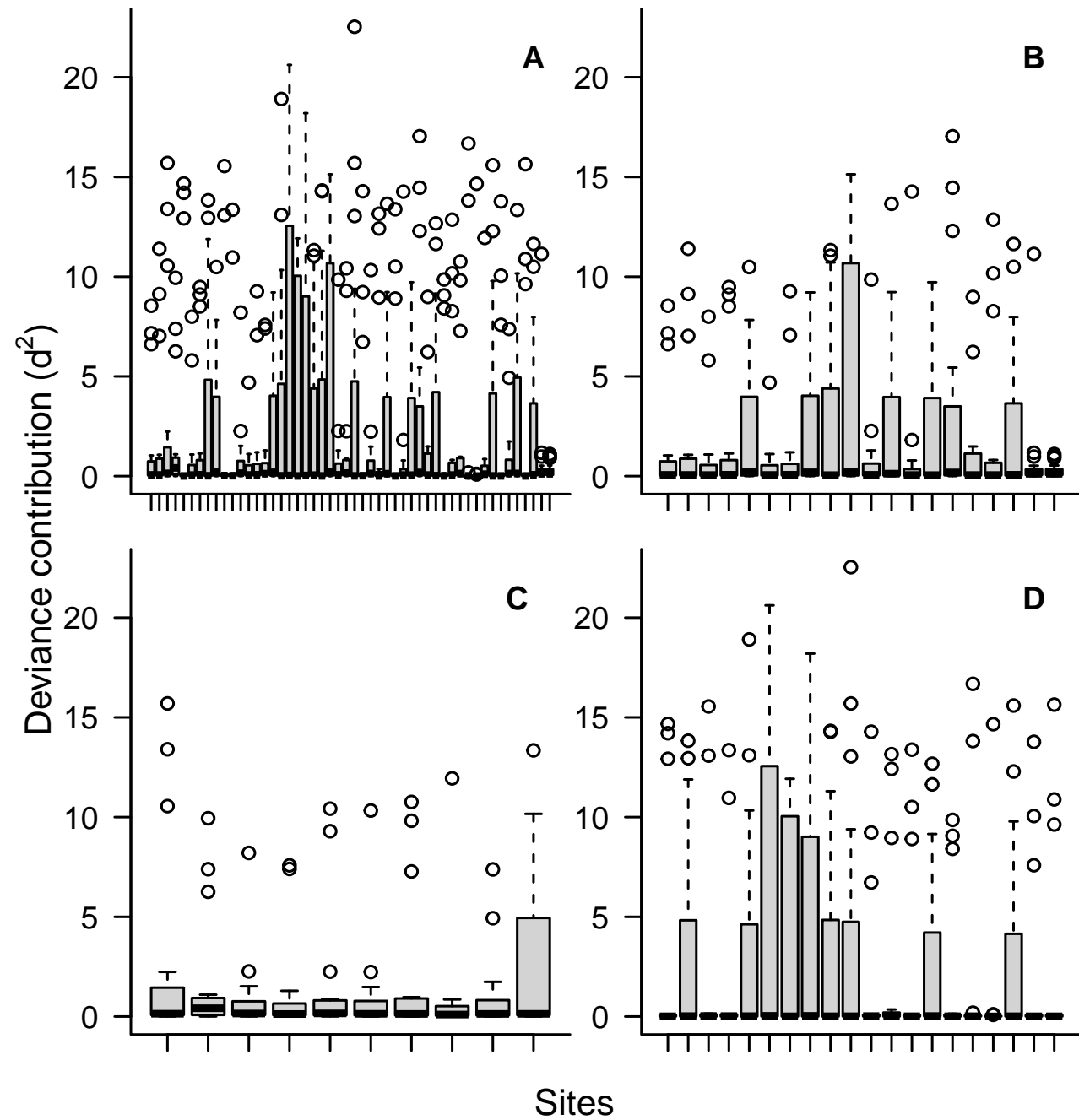

Supplement: Supplementary file 1 — Appendices S1–S6: ece372502‐sup‐0001‐AppendicesS1‐S6.zip. [file ECE3-15-e72502-s001.zip › Appendix_FigureA3.2.pdf]

Deviance contribution ( $d^2$ )

**A**

**C**

**B**

**D**

Taxon

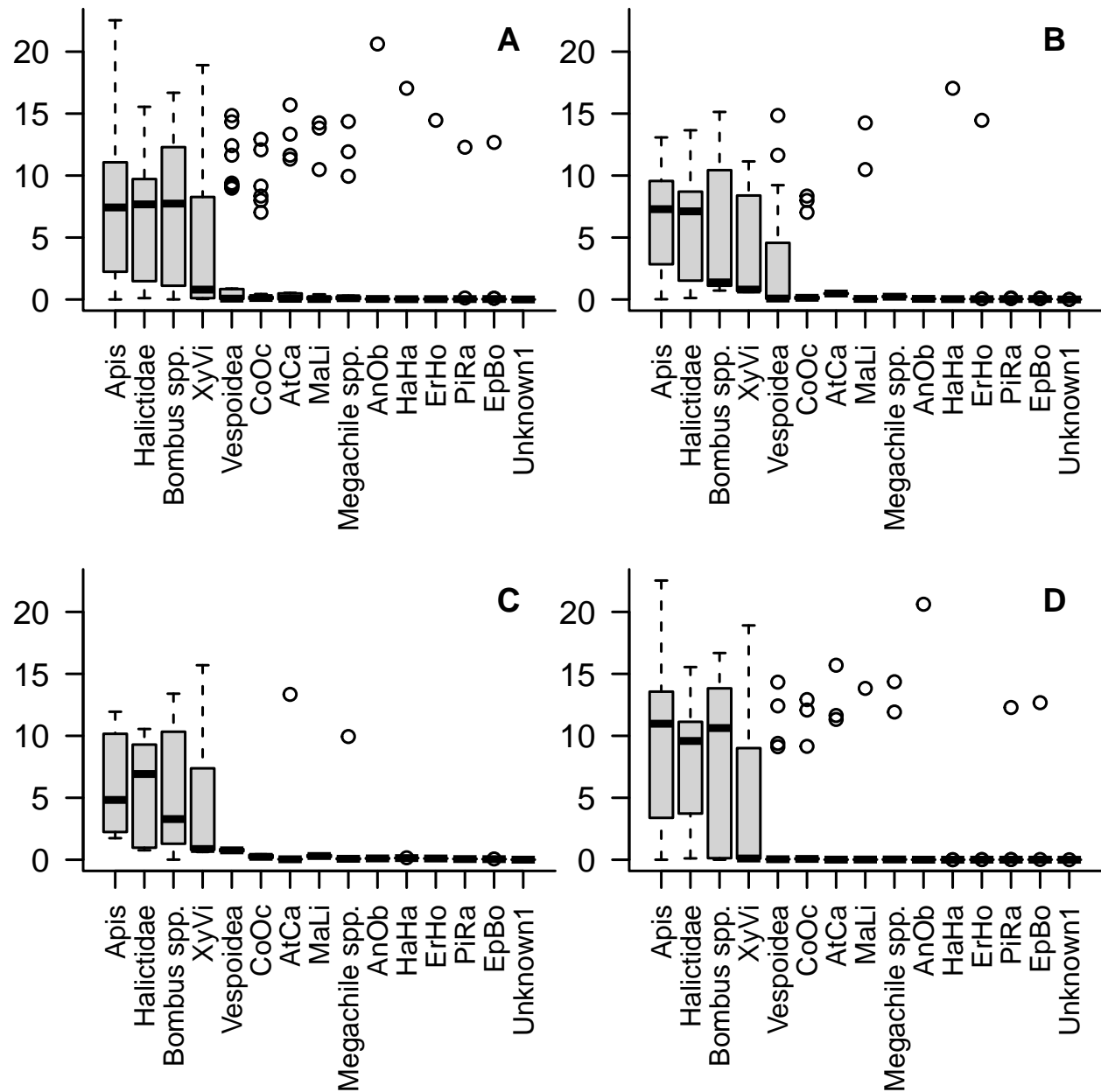

Supplement: Supplementary file 1 — Appendices S1–S6: ece372502‐sup‐0001‐AppendicesS1‐S6.zip. [file ECE3-15-e72502-s001.zip › Appendix_FigureA3.3.pdf]

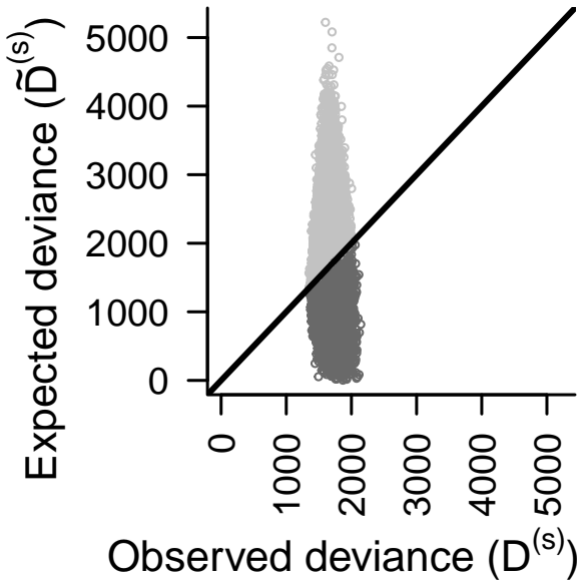

Supplement: Supplementary file 1 — Appendices S1–S6: ece372502‐sup‐0001‐AppendicesS1‐S6.zip. [file ECE3-15-e72502-s001.zip › Appendix_FigureA3.1.pdf]
